# Spatial transcriptomics differences of histopathological growth patterns in colorectal cancer liver metastases

**DOI:** 10.1038/s41416-026-03567-y

**Published:** 2026-07-28

**Authors:** Maria Escriva Conde, Axel Andersson, Peter Vermeulen, Mats Nilsson, Hanna Nyström

**Affiliations:** 1 Science for Life Laboratory, Department of Biochemistry and Biophysics, Stockholm University, Solna, Sweden; 2 Department of Diagnostics and Intervention, Surgery, Umeå University, Umeå, Sweden; 3 Translational Cancer Research Unit, Ziekenhuis aan de Stroom, Campus Augustinus, Antwerp, Belgium; 4 Wallenberg Centre for Molecular Medicine, Umeå University, Umeå, Sweden

**Keywords:** Transcriptomics, Cancer microenvironment, Tumour heterogeneity, Metastasis

## Abstract

**Background:**

Histopathological growth patterns (HGPs) have emerged as prognostic and predictive biomarkers in colorectal liver metastases (CLM). The desmoplastic/encapsulating HGP (EHGP) is associated with improved survival after surgery, whereas the replacement HGP (RHGP) is associated with poorer outcomes. However, HGPs can only be reliably assessed postoperatively, limiting their use as preoperative biomarkers. A deeper molecular understanding of HGPs could inform biomarker development and therapeutic strategies, including approaches to promote EHGP.

**Methods:**

We applied in situ sequencing (ISS), an image-based spatial transcriptomics method, to map RNA expression in CLM tissue. Two custom gene panels (150 and 175 genes) were analysed in a chemonaïve cohort of resected CLM tissue from 19 patients with EHGP and RHGP.

**Results:**

Distinct molecular programmes characterised the two patterns. RHGP was associated with damaged *SAA1*^+^ hepatocytes, *KRT18*^+^ neoplastic cells, and bifunctional hepatocyte-cholangiocyte cells. EHGP showed interferon-γ signalling, cytotoxic T-cell infiltration, and a fibrotic capsule with zonation-specific features: hepatic stellate cell activation, angiogenesis, and immune recruitment on the liver-facing side, and cancer-associated fibroblasts on the tumour-facing side.

**Conclusions:**

These differences imply that growth patterns emerge from tumour-host driven interactions. They reveal new molecular features of the metastatic niche. These insights may inform the search for novel treatment strategies.

## Background

Colorectal cancer (CRC) is the third leading cause of cancer-related deaths worldwide [1]. A major contributor to this mortality is the development of metastatic disease, which significantly reduces long-term survival. Over 30% of CRC patients will develop colorectal liver metastases [2]. For patients with resectable colorectal liver metastases (CLM), 5-year overall survival exceeds 50% [3], and 10-year survival rates above 24% have been observed, with approximately 20% disease-free survival [4]. To improve outcome, several prognostic and predictive traits for CLM have been proposed.

One promising avenue is using histopathological growth patterns (HGPs) as biomarkers. Two main types have been identified: the desmoplastic HGP (DHGP) and the replacement HGP (RHGP). DHGP is characterised by a fibrotic rim that separates tumour cells from liver tissue, whereas RHGP involves tumour cells infiltrating the liver parenchyma, replacing hepatocytes, and co-opting the native vasculature [5]. These patterns are not merely morphological but are strongly associated with clinical outcomes, including survival following liver surgery [6, 7] and response to therapy [8, 9].

Despite their clinical relevance, HGPs can currently be assessed only in resected tissue, limiting their utility as preoperative biomarkers. To address this, recent studies have explored the use of medical imaging techniques to infer HGPs noninvasively before surgery [10–13]. Parallel efforts have focused on identifying molecular markers that could act as surrogates for HGPs or shed light on the biological mechanisms driving their development [14–20]. These molecular studies show that HGPs are associated with distinct transcriptomic profiles. RHGP is linked to increased tumour cell motility [8, 14], immunosuppression [17], neutrophil enrichment [15], metabolic reprogramming [16, 18], cell proliferation [20], poor response to anti-angiogenic therapy [8], and poor survival following liver surgery [7]. By contrast, DHGP has been associated with immune infiltration, angiogenesis, a favourable immune microenvironment [16, 20], and better overall survival following liver surgery [7].

Recent evidence suggests that DHGP is not purely tumour-driven desmoplasia [19]. Instead, it is proposed to arise from an injury-induced wound-healing response in the liver, suggesting that the desmoplastic rim is a host-driven reaction that stalls tumour invasion. This has led to the term encapsulated HGP (EHGP) [21], which we will use in this article.

Collectively, these insights support the emerging view that HGPs are not hardwired genetically but are likely governed by tumour-microenvironment cues. A deeper molecular understanding may inform biomarker development and, ultimately, therapeutic strategies (potentially including approaches to promote EHGP over RHGP). However, the mechanisms underlying these HGPs remain incompletely understood. To address this knowledge gap, we apply a spatially resolved, highly multiplexed targeted transcriptomic method, in situ sequencing [22, 23], to directly compare the molecular landscapes of EHGP and RHGP CLM lesions. In situ sequencing (ISS) enables high-resolution gene expression mapping, providing novel insights into the biology of HGPs.

Our analyses revealed molecular features of the two growth patterns: RHGP lesions contained damaged *SAA1*⁺ hepatocytes and cells with cholangiocyte-like or intermediate hepatocyte-cholangiocyte features in the tumour region, whereas EHGP lesions showed strong interferon-γ signalling, cytotoxic T-cell infiltration, and a zonated fibrotic capsule enriched in hepatic stellate cell (HSC) signatures, including *CXCL12* expression spatially adjacent to *CXCR4*⁺ cytotoxic T-cells. Our observations suggest that HGPs likely arise from specific tumour-host interactions. Together, these findings deepen our understanding of HGPs and may contribute to future treatment development and patient stratification.

## Methods

**Fig. 1:**
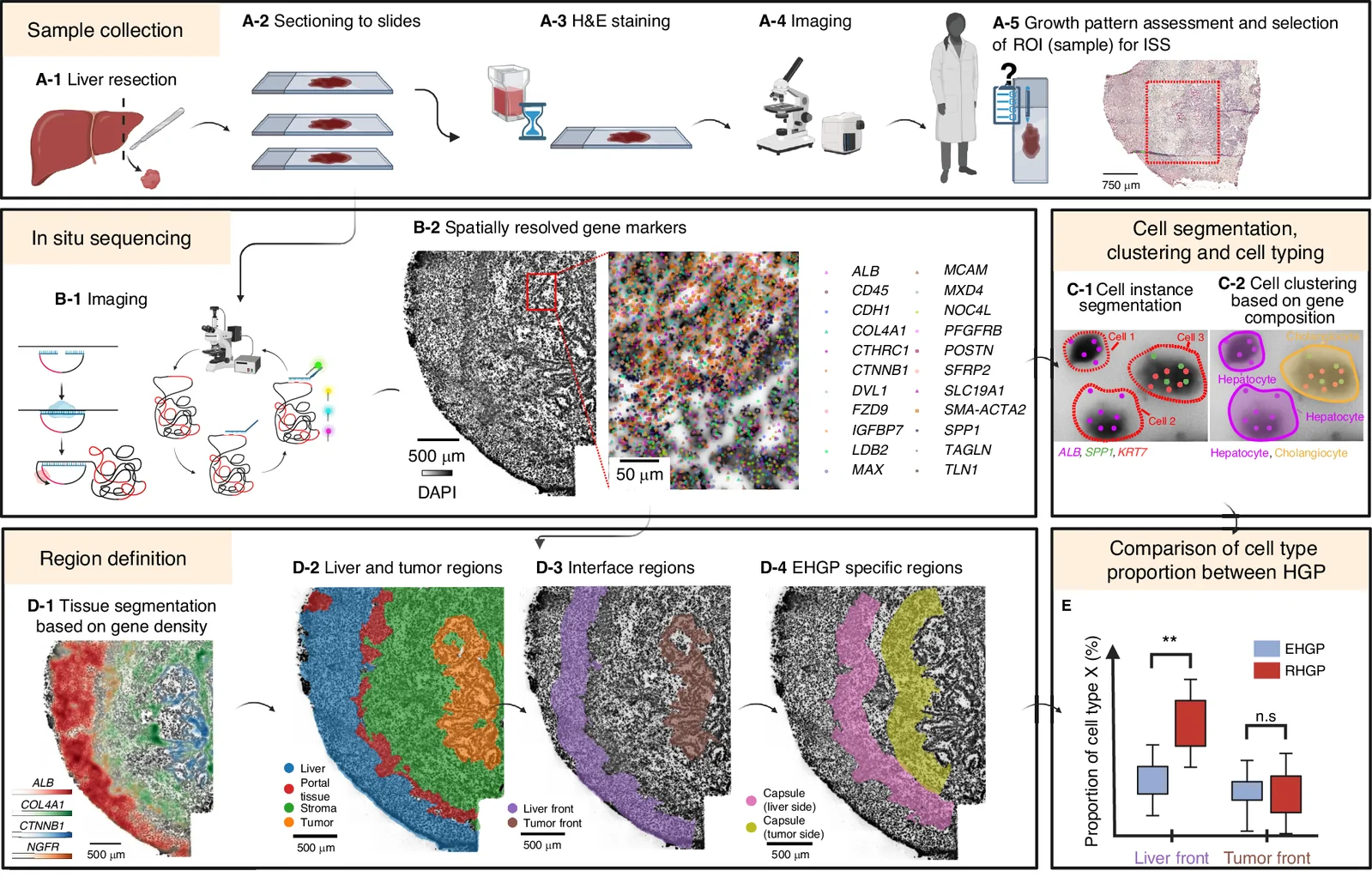
Overview of key methodological steps in the study. Liver tissue was resected from patients with CLM (**a-1**). The tissue was sectioned into slides (**a-2**), stained with hematoxylin and eosin (HE; **a-3**), and imaged (**a-4**). A pathologist classified the HGP and selected the ROI (‘samples’) for ISS (**a-5**). For each metastasis, two sectioned slides were used for ISS staining (**b‑1**) with two gene panels (**b‑2**) and imaged within the selected ROI. ISS generated spatial markers (reads) representing individual mRNA molecules, labelled by their corresponding genes, along with a nuclear stain (DAPI) for spatial context. These data were then used for cell segmentation, assigning reads to individual cells (**c-1**). Segmented cells were clustered based on gene composition (**c-2**) into clusters representing cell types. Local gene densities together with nuclei morphology (**d-1**) were used to classify the tissue into four regions (**d-2**) representing liver, portal tissue, stroma, and tumour. Two additional distance-based regions were defined (**d-3**): the liver front and tumour front regions, extending 300 μm into the liver and tumour regions. Lastly, two regions used only for EHGP samples were defined: ‘capsule (liver side)’ and ‘capsule (tumour side)’, each covering 300 μm of tissue outwards from the liver and tumour regions, respectively (**d-4**). The proportion of cell types was analysed across regions and compared between growth patterns (**e**). All panels were created in BioRender (2025) https://BioRender.com/.

The study was conducted in several steps, combining experimental and computational approaches to analyse spatial gene expression patterns. Figure 1a–e summarises these steps, and each is described below.

### Sample collection

First, metastatic tissue was collected and processed before spatial analysis (Fig. 1a-1). This study used fresh frozen tissue from patients undergoing liver surgery for CLM between 2017 and 2019 at the Norrlands Universitetssjukhus, Sweden. After surgery, the resected metastasis was immediately snap-frozen in liquid nitrogen and stored at −80 °C until analysis. All patients provided written informed consent to participate in the tissue biobank of Region Västerbotten, Sweden.

The inclusion criteria were: (i) patients who had not received preoperative oncological treatment for at least six months before liver surgery, and (ii) patients who underwent atypical liver resections to minimise the ischaemic time of the resected tissue. Exclusion criteria included: (i) patients who received recent (<6 months) preoperative oncological therapy; (ii) patients who underwent major liver surgeries (e.g. hemihepatectomy or extended hemihepatectomy), and (iii) samples that did not meet the quality requirements for the assessment of HGP, such as lacking an intact tumour-liver interface or viable tumour cells in the section.

**Fig. 2:**
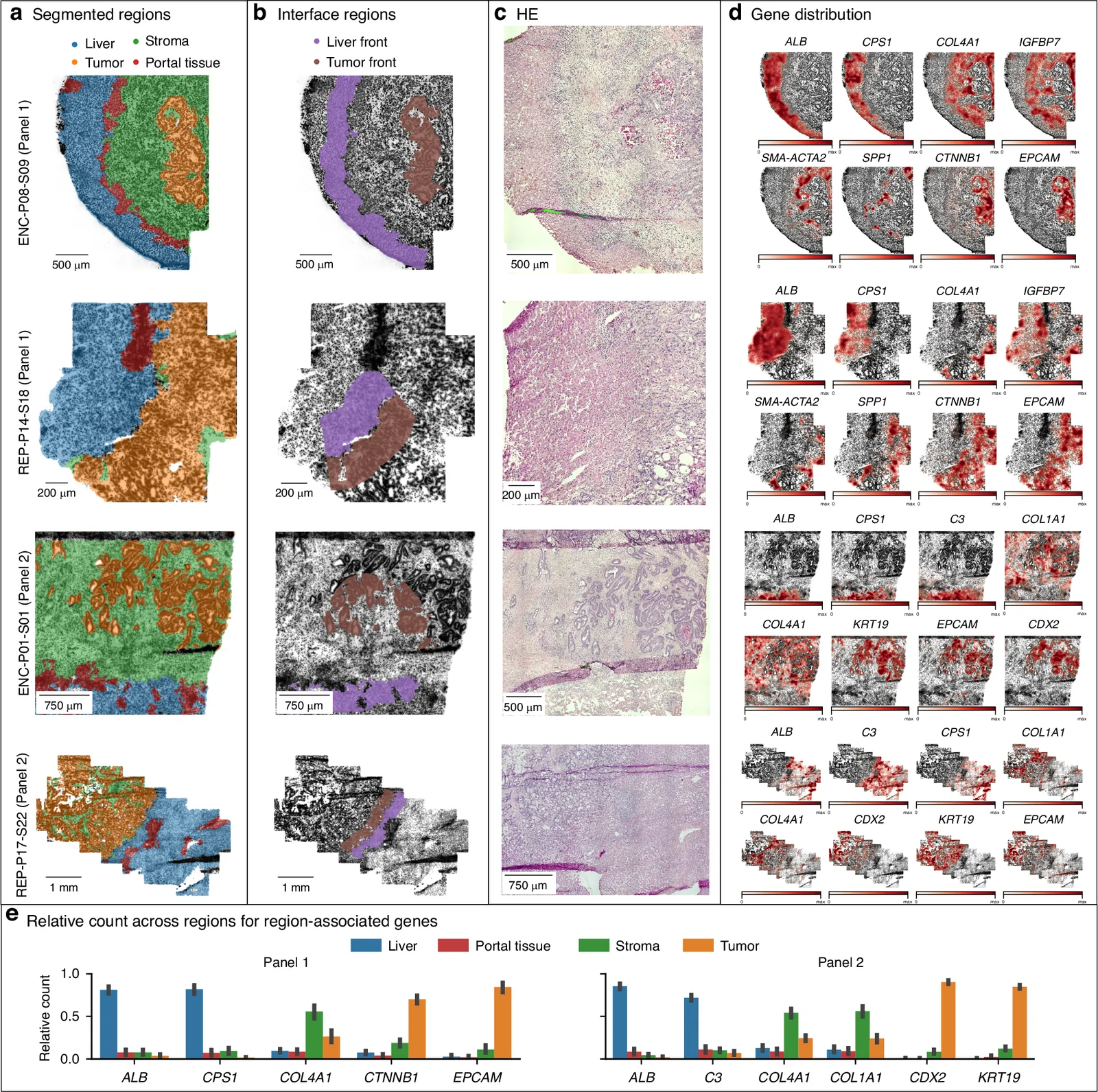
Spatial transcriptomic data from CLM samples were segmented into hepatocyte-rich liver, portal, stromal and tumour regions. **a** DAPI-stained image together with overlaid segmented regions, highlighting hepatocyte-rich liver, stroma, tumour and portal-type tissue. **b** Two interface regions: the liver front, representing the first 300 µm of liver tissue from the tumour-liver interface (RHGP) or liver-capsule interface (EHGP); and the tumour front, representing the first 300 µm of tumour tissue from the tumour-liver interface (RHGP) or tumour-capsule interface (EHGP) (**c**). HE-stained histological images for a morphological context. Each row corresponds to a different sample (**d**). Density maps showing spatial expression patterns of selected genes, providing molecular context. **e** Relative counts of selected genes across regions for Panel 1 (left) and Panel 2 (right), showing enrichment of *ALB*, *CPS1* and *C3* in liver, *COL4A1* and *COL1A1* in stroma, and *CTNNB1*, *EPCAM*, *CDX2* and *KRT19* in tumour.

**Fig. 3:**
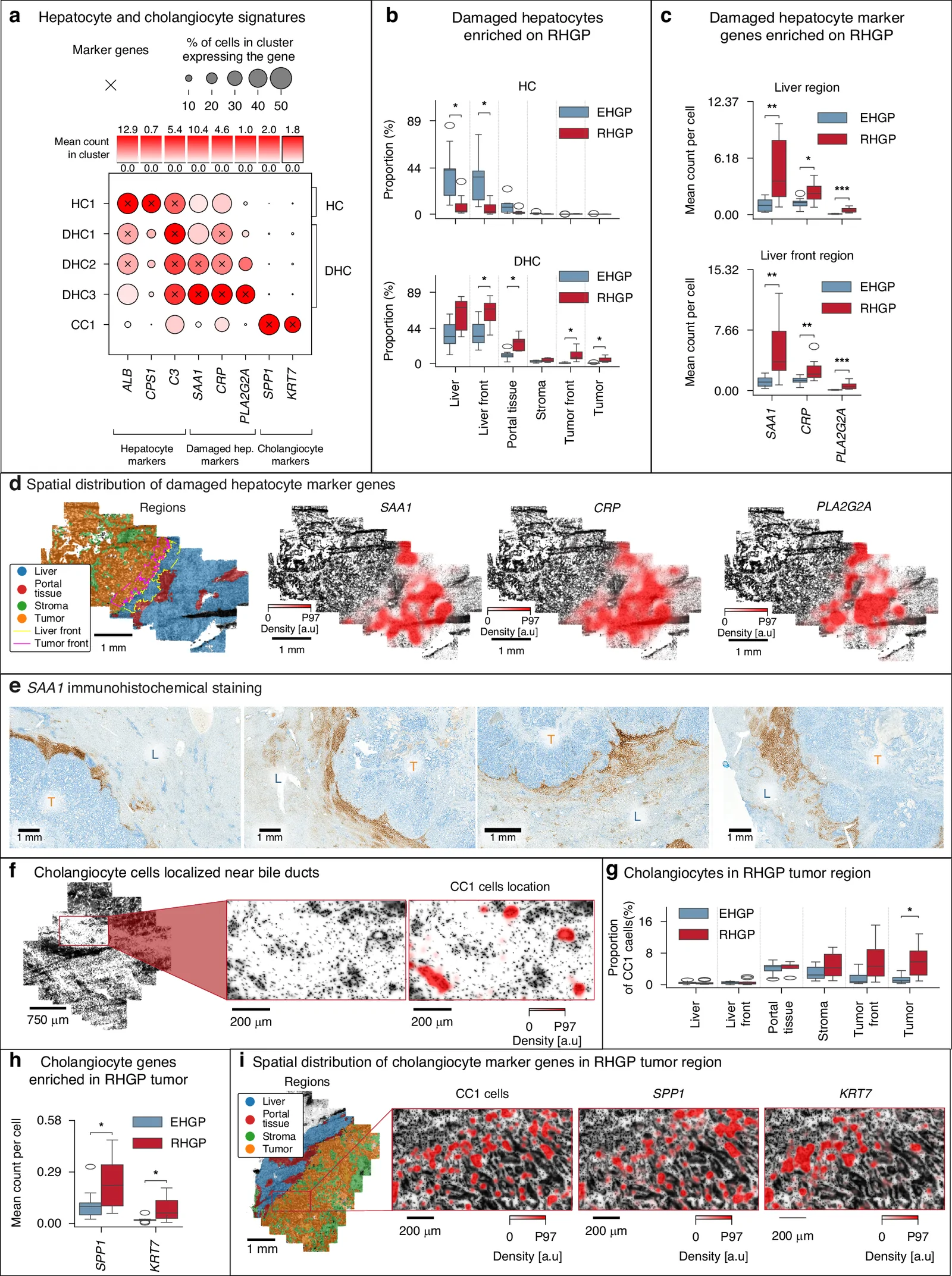
RHGP samples exhibit damaged hepatocytes at the liver front and cholangiocyte cells in the tumour region. **a** Dotplot showing average gene count of selected genes for four different cell clusters annotated as hepatocytes (HC1), damaged hepatocytes (DHC1–3), and cholangiocytes (CC1). **b** Boxplots comparing the proportion of hepatocytes (HC) and damaged hepatocytes (DHC) across different regions. **c** Comparison of mean *SAA1*, *CRP*, and *PLA2G2A* count per cell in the liver and liver front regions of EHGP- and RHGP samples. Expression of all three genes was significantly higher in RHGP samples. **d** Read density of genes associated with damaged hepatocytes (RHGP sample). **e** Representative immunohistochemical staining in four RHGP samples. **f** DAPI staining of an EHGP sample showing CC1 cells aligned with bile ducts, recognisable by their ring-like nuclear arrangement. **g** Boxplots comparing the proportion of these cells between the two growth patterns in different regions. A larger proportion of CC1 cells was found on RHGP samples in the tumour region. **h** Comparison of mean *SPP1* and *KRT7* count per cell in the tumour region of EHGP- and RHGP-labelled samples. Both genes were upregulated in the tumour region in RHGP samples. **i** Spatial density of CC1 cells, *SPP1* and *KRT7* in an RHGP sample, showing cholangiocyte-associated signals near tumour cells.

**Fig. 4:**
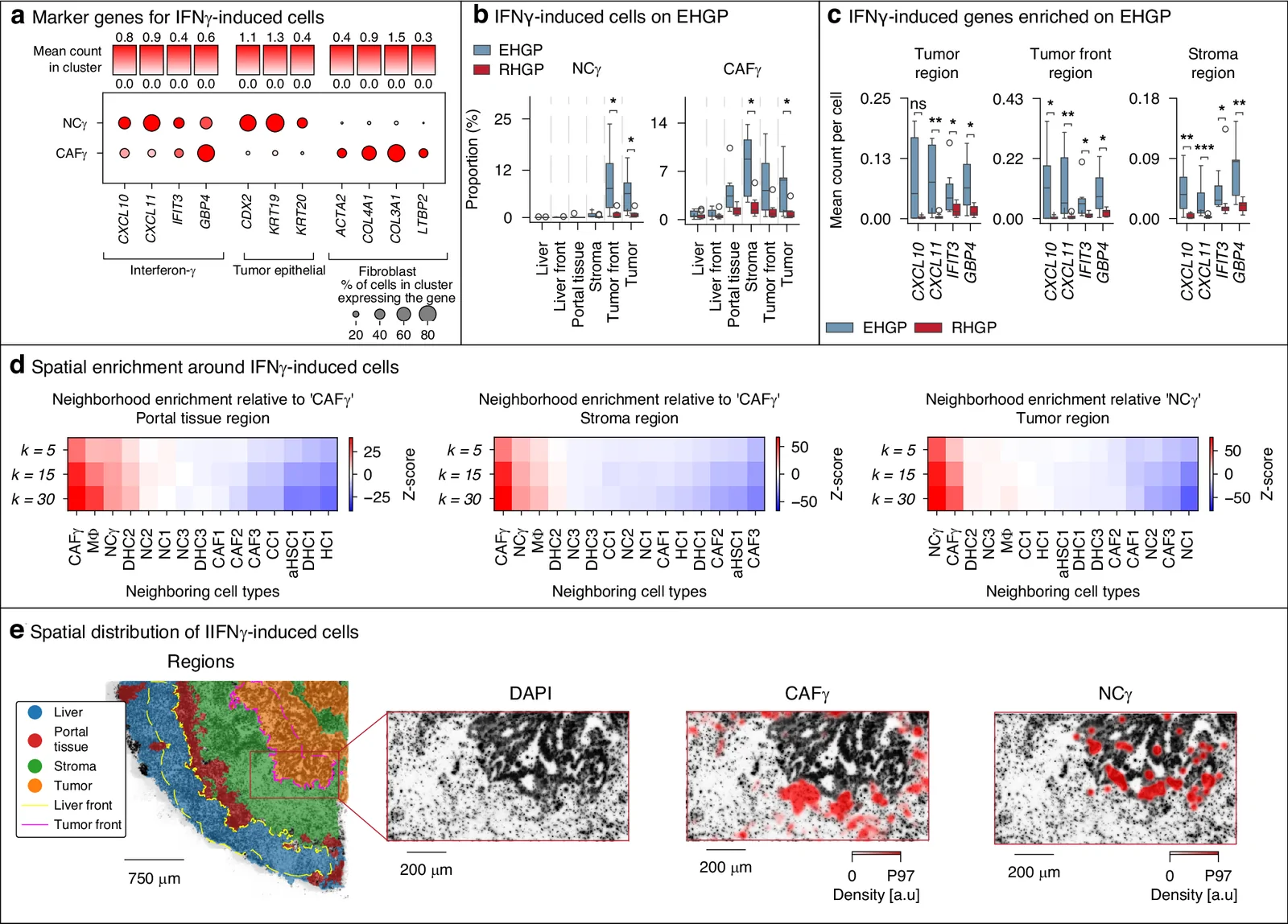
Increased interferon-γ signalling in samples with an EHGP. **a** Dotplot highlighting gene expression for two interferon-γ-induced cell clusters: the neoplastic cell cluster (NCγ), and cancer-associated fibroblasts (CAFγ). **b** Boxplots comparing the proportion of these cells between the two growth patterns in different regions. A larger proportion of NCγ cells were found on RHGP-labelled samples in the tumour and tumour front region, whereas a larger proportion of CAFγ cells were found in the stroma region on EHGP-labelled samples. **c** Comparison of mean read count of IFN-associated genes (*CXCL10*, *CXCL11*, *IFIT3*, *GBP4*) per cell in stromal, tumour, and tumour-front regions between EHGP- and RHGP-labelled samples. Expression was higher in EHGP samples, supporting an IFNγ-enriched microenvironment. **d** Neighbourhood enrichment scores of clusters around the encapsulating-dominated IFNγ-associated cells. The scores are computed in three regions: portal tissue (top), stroma region (middle), and tumour region (bottom). An up-regulated enrichment score between CAFγ and MΦ was observed across all regions. **e** Spatial distribution of IFNγ cells, with CAFγ cells located in the tumour-adjacent stroma, and NCγ cells in the tumour pseudocrypts.

**Fig. 5:**
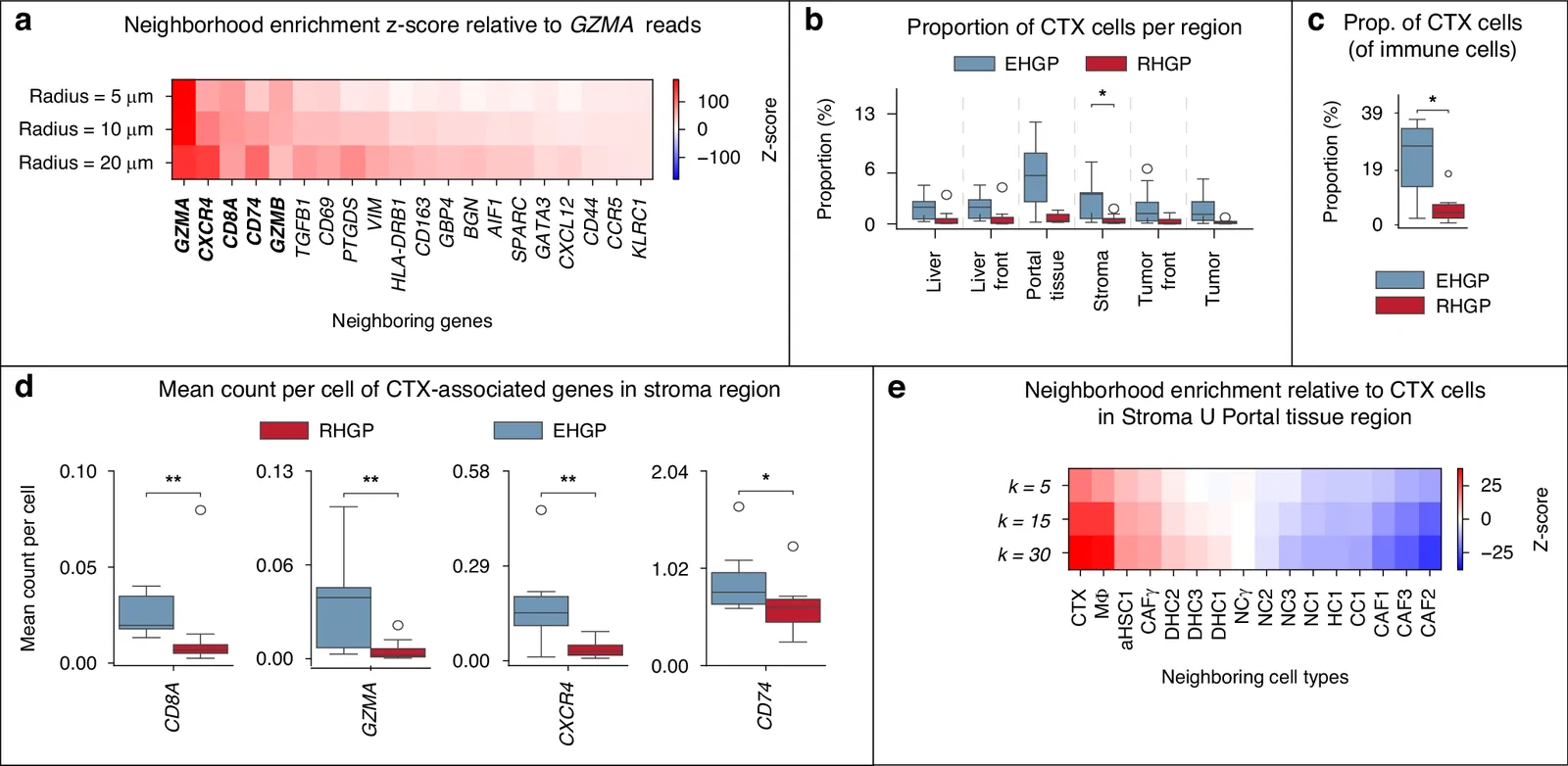
Identification and characterisation of cytotoxic T-cells enriched in EHGP samples. **a** Neighbourhood enrichment test (radii of 5, 10, and 20 μm) around *GZMA* reads revealed co-enrichment of other cytotoxic T-cell (CTX) related genes, including *3*, *CXCR4*, and *CD74* at cellular scales. CTX cells were more abundant in the stroma of EHGP samples, both in absolute proportion (**b**) and relative to immune cells (CTX and MΦ; **c**). **d** Mean read counts (across cells) of *GZMA*, *CD8A*, and *CXCR4* in the stroma region were higher in EHGP compared to RHGP samples. **e** Neighbourhood enrichment (*k* = 5, 15, and 30 neighbours) in combined stroma and portal regions showed CTX cells were frequently adjacent to MΦ macrophages, CAFγ fibroblasts, and aHSC1 hepatic stellate cells. *CXCL12* expression in aHSC1 cells supports potential recruitment of CTX via the *CXCR4*–*CXCL12* axis.

The cohort included 19 patients, each contributing with one metastasis (Fig. 1a-1). From each metastasis, ten consecutive 4 µm tissue sections were prepared using a Leica cryotome (Fig. 1a-2). Hematoxylin–eosin (HE) staining was first performed in one section (Fig. 1a-3-4). In this HE‑stained section, one or two regions of interest (ROIs; ~5 mm²) with distinct growth patterns were identified by two observers (PV and HN) following international guidelines (Fig. 1a-5).

ISS with two gene panels was then performed separately on two sequential sections, and imaging was restricted to the selected ROIs to investigate potential differences between the HGPs. Throughout the manuscript, we refer to each ROI used for ISS as a ‘sample’*.*

In a few cases, two samples originated from the same metastasis. When these samples shared the same HGP, their measurements were averaged to avoid inflating the number of independent observations. In the two cases where samples from the same metastasis represented different HGPs (*n* = 2), each was treated as an independent observation in downstream analyses. The mapping between patients, metastases, samples, and growth patterns is summarised in Supplementary Table 1.

### In situ sequencing

Next, ISS was used to spatially map the gene expression within EHGP and RHGP labelled samples (Fig. 1b-1). For each sample, two sequential slides were processed for the direct RNA ISS method [23] following the detailed protocol and image-analysis pipeline described by Andersson et al. [24].

Two panels, Panel 1 (150 genes) and Panel 2 (175 genes), were used for each sequential slide. The two panels shared 33 gene targets. The overlapping genes were intentionally included to assess the reproducibility of expression patterns across experimental runs. The gene panels, the L-probes, and the detection oligo sequences per gene are detailed in Supplementary Table 2.

Panel 1 was designed based on an initial analysis of bulk RNA sequencing data, later published by Latacz et al. [20], aiming to identify differences between EHGP and RHGP. Panel 2 was motivated by insights gained from the initial Panel 1 analysis [24] and reflects a shift in focus toward the tumour microenvironment. A single unified panel was not used due to the sequential evolution of the study design.

ISS analysis was previously performed on 10 EHGP samples using Panel 1 [24]; here, we reanalysed the previous 10 EHGP samples and added 16 RHGP samples (Supplementary Table 1). One RHGP sample was excluded after ISS due to poor staining quality, resulting in 25 samples from 19 patients.

Panel 2 was designed to capture cancer-associated fibroblast (CAF) heterogeneity across growth patterns and included 10 EHGP and 16 RHGP samples (Supplementary Table 1). Four RHGP samples were excluded after ISS from further analysis due to poor staining quality, resulting in 22 samples from 17 patients.

The ISS experiments generated millions of reads of the targeted genes, each representing an individual mRNA molecule with a spatial (2D) position and a gene identity label (Fig. 1b-2). Cross-panel integration was not performed. Data from Panel 1 and Panel 2 were analysed independently, and cell-type definitions were derived separately within each panel. No cell-type annotation relied on markers split across panels, and gene expression data from the two panels were not merged. Panel 1 and Panel 2 were used to address complementary biological questions.

### Cell segmentation

ISS data can be analysed similarly to single-cell data by assigning reads to cells [25], effectively creating a cell-by-gene matrix. Thus, the next step of the study was to segment cells (Fig. 1c-1). CellPose V.3 (Cyto 3 model, RRID:SCR_021716) [26] was used to detect nuclei in DAPI-stained images obtained in the ISS experiment. The detected nuclei seeded a probabilistic model (Baysor) [27] which assigned mRNA reads to cells, creating the cell-by-gene matrix, stored as an AnnData object (RRID:SCR_018209). The Baysor nuclei-segmentation confidence parameter was set to 0.5, and the spatial scale was set to 45 pixels (7.2 μm).

### Single-cell analysis

#### Clustering

Next, clustering was used to dissect the cell-by-gene data into a few biological components representing cellular types (Fig. 1c-2). Lowly expressed genes (<5000 total number of reads across all samples) and low-quality cells (<8 total number of reads) were filtered out. Cells were normalised by total read count across genes. The normalised counts per gene were scaled by subtracting the mean and dividing by the standard deviation (capped at a maximum value of 100) across all cells. Principal component analysis (PCA) was performed, retaining the top 10 components, which corresponded roughly to the elbow point of the explained-variance curve (Supplementary Fig. 1).

A *k*-nearest neighbours’ graph (*k* = 30) was constructed using PCA embeddings, and community detection was performed via the Louvain algorithm at a resolution of 1.25 [25]. Louvain clustering is non-deterministic. It depends on the order of the nodes in the graph. To make sure the clustering was stable, it was repeated 100 times with different orderings of the nodes. Mean gene expression profiles were computed for each cluster in each repetition and compared across repetitions using Pearson correlation. Clusters with a correlation >0.95 were considered equivalent. Clusters appearing in at least 75 repetitions were labelled as *stable* and retained. Hierarchical clustering, with single linkage and a distance threshold of 0.05 based on 1 - correlation, was used to merge the stable and correlated clusters.

Final cluster assignments were determined via majority voting: for each cell, the number of times it was assigned to each merged cluster across repetitions was counted, and the cluster with the highest frequency was assigned as the final label.

#### Marker genes

Marker genes were identified using a tie-corrected one-sided Mann-Whitney U-Test with ScanPy V1.10.0 (RRID:SCR_018139), comparing raw gene counts within each cluster to all other cells. A gene was considered a marker if it had (i) significantly higher expression in the cluster (*p* < 0.05, Benjamini-Hochberg corrected), (ii) a log-fold change ≥ 2, and (iii) expression in at least 20% of the cluster’s cells.

### Region definition

Thereafter, tissue regions were defined to analyse clustered cells in a spatial context (Fig. 1d-1). The tissue was divided into four regions: liver, tumour, portal tissue, and stroma, using QuPath’s (V.0.5.1) Pixel Classifier (RRID:SCR_018257; Fig. 1d-2).

Gene densities were estimated using a Gaussian kernel (*σ* = 10 µm), producing a multi-channel image where each channel represented the density of a given gene. Dimensionality was reduced via non-negative matrix factorisation (15 factors; sklearn V.1.5.0, RRID:SCR_019053) to generate ‘meta-gene’ channels, which were combined with the nuclear stain image and exported as an OME-TIFF for classification in QuPath.

The classifier was trained on small manually annotated regions in the OME-TIFF image. The training regions were selected based on nuclear morphology, characteristic gene expression, and HE morphology, by a computer scientist with guidance from a pathologist. Characteristic genes guiding selection included albumin for liver, collagens for stroma, *CTNNB1* and *CDX1* for tumour, and *NGFR* and *PTGDS* for portal tissue. The trained classifier was applied to full sections, and segmentation was refined by manual removal of artifacts such as tissue folds. The final segmentations were validated by a pathologist.

Two interface regions were also defined: the liver front and tumour front, each covering 300 µm from manually selected liver or tumour borders (Fig. 1d-3). In replacement samples, borders were placed where hepatocytes and tumour cells directly interacted, whereas in EHGP samples, liver and tumour borders were defined separately due to the intervening capsule.

Finally, two EHGP-specific regions were defined: the ‘capsule (liver side)’ and ‘capsule (tumour side)’, each covering 300 µm of stroma and portal tissue directly outside the liver or tumour region, respectively (Fig. 1d-4).

### Comparing cluster proportions between growth patterns

The clustered cells and defined regions were used to analyse differences between growth patterns. For each sample, the proportion of cells belonging to the different clusters was calculated in each region. The proportions were then compared using Tukey boxplots (Fig. 1e). Significant differences in cluster proportions between the growth patterns were determined by the two-sided Mann-Whitney U-Test with Benjamini-Hochberg correction (SciPy V1.14.0; RRID:SCR_008058). When two samples from the same patient shared the same HGP, their cluster proportions were averaged to avoid repeated sampling.

### Mean read count comparison

To assess whether a given gene was differentially expressed between the growth patterns within a selected region, we (i) calculated the mean read count per cell for that gene within the region for each sample and (ii) compared EHGP and RHGP using a two‑sided Mann-Whitney U‑test (SciPy v1.14.0, RRID:SCR_008058). When two samples from the same patient shared the same HGP, their mean values were averaged to avoid repeated sampling.

To account for variation in tissue quality, we additionally performed the analysis with a sample‑wise normalisation. For each sample, the gene’s mean read count was divided by the sample’s mean total read count (across all genes) and then multiplied by a constant (the global mean total read count across all cells in all samples) to preserve the original data scale.

### Neighbourhood enrichment analysis

To quantify spatial relationships between cell types and other labels, we performed a neighbourhood enrichment analysis. Neighbourhood enrichment scores were computed using Squidpy [28]. For each pair of labels (e.g. cell types or genes), observed neighbour counts were compared against a null distribution generated by 1000 label permutations. Positive and negative *z*‑scores were interpreted as enrichment and depletion, respectively. Analyses were repeated across spatial scales by varying the neighbourhood‑definition parameters, including the number of nearest neighbours (*k*) or a fixed distance radius (*r*).

### Capsule zonation quantification

It has recently been established that the biology of the capsule in EHGP differs between the tumour and liver sides [19, 24]. To explore the zonation of gene expression across the capsule in EHGP, read counts per area were calculated for each gene in four regions: liver front, capsule (liver side), capsule (tumour side), and tumour front. For each gene, these values were normalised by the sum across all regions, resulting in a relative count per region. Average relative counts across EHGP samples were visualised in a heatmap.

### Protein-level validation

After completing the ISS analysis, protein‑level assays were performed to validate selected transcriptomic findings.

#### Immunohistochemical staining

To validate SAA1 expression in hepatocytes, we performed immunohistochemical (IHC) on eleven RHGP samples, classified based on HE slides.

Paraffinated tissue from patients undergoing resection from CLM was sectioned (4 µm; Norrlands Universitetssjukhus, 2015–2020; written informed consent) and stained using the Ventana Benchmark Ultra automated immunostainer (Ventana Medical Systems, Tucson, AZ, USA) using the rabbit monoclonal anti-serum Amyloid A antibody (clone EP11592-92, ab190802, Abcam, Camebridge, UK; 1:300 dilution) diluted in antibody diluent buffer (251-018, Ventana Medical Systems, Tucson, AZ, USA). The staining protocol included an antigen retrieval step with CC1/pH 9 buffer (ULTRA CC1, 950-224, Ventana Medical Systems, Tucson, AZ, USA), incubation with the primary antibody for 32 min at room temperature (RT) and Chromogenic detection was achieved using the UltraView Universal DAB Detection Kit (760-500, Ventana Medical Systems, Tucson, AZ, USA), and subsequently counterstained with hematoxylin (790-2208, Ventana Medical Systems, Tucson, AZ, USA). Whole-slide images were visualised with QuPath 0.6.0.

#### Multiplex immunofluorescence (COMET; Lunaphore)

Multiplex immunofluorescence was used to validate the presence of potential bifunctional hepatocyte-cholangiocyte populations in RHGP tissue sections. Tissue samples were obtained from the Biobank of the Oncology Centre Antwerp at the Ziekenhuis aan de Stroom (ZAS), Antwerp, Belgium, and provided as FFPE tissue microarray (TMA) slides comprising eight cores. Sequential immunofluorescence analysis was performed on the COMET platform (Lunaphore Technologies). TMA slides were deparaffinized and subjected to heat‑induced epitope retrieval according to the manufacturer’s recommendations, then loaded onto the COMET Stainer and processed using the standard sequential staining workflow. Samples underwent iterative cycles of primary antibody incubation followed by fluorophore‑conjugated secondary antibodies, with imaging in each round.

The antibody panel consisted of five markers (*KRT7, SPP1, CRP, HSA, CDX2*). Antibody details (antibody name, target, lot, clone, host species, manufacturer, catalogue number, dilution factor, acquisition channels) are provided in Supplementary Table 3.

Imaging outputs from each staining cycle were automatically captured, stitched, and aligned by the COMET software. Images were generated and reviewed using the Lunaphore Image Viewer after background fluorescence subtraction.

##### Ethical approval and consent to participate

This study was conducted in compliance with the Helsinki Declaration. Procedures complied with national regulations and were approved by the relevant ethics/biobank oversight bodies as detailed below. Sweden (Umeå/Region Västerbotten). Patients undergoing liver surgery for CLM at Norrlands Universitetssjukhus (2015–2020) were enroled under approval from the Swedish Ethical Review Board (DNR 2019‑05690). Written informed consent was obtained from all participants for inclusion in the Region Västerbotten biobank. Fresh‑frozen sections from these lesions were used for ISS and for IHC staining for SAA1. Belgium (Antwerp/ZAS Biobank). Human CLM samples for multiplex immunofluorescence (COMET) were obtained from the Biobank of the Oncology Centre Antwerp at Ziekenhuis aan de Stroom (ZAS), registered with the Federal Agency for Medicines and Health Products of Belgium (BB190028), and approved by the Ethical Review Board (ERB) of ZAS on 11 February 2019. The liver metastasis tissue fragments represent remnant clinical tissue. stored under an opt‑out framework; under Belgian regulations, specific informed consent is not required for biobanking such remnant material provided an opt‑out is in place (as at ZAS). The ERB of ZAS further approved research use of these specimens (200609ACADEM, 2 June 2020).

## Results

### Spatial transcriptomics mapping and region annotations

We spatially mapped gene expression across 26 CLM samples from 19 chemo-naïve patients using ISS. Each sample displayed either an EHGP or an RHGP. To explore spatial features in detail, we divided each sample into regions representing the liver, tumour, stroma, and portal tissue. In addition, we defined two interface regions, liver front and tumour front, covering the first 300 µm from the boundary of the liver and tumour regions. These interfaces allowed us to specifically target areas where tumour-hepatocyte interactions occur, depending on the underlying HGP. All regions were visually validated by a pathologist. Figure 2a, b displays the segmented regions for four representative samples in the study. Alongside these are histological images (Fig. 2c) and gene density maps (Fig. 2d), providing molecular and morphological references. Additionally, all images, regions, and gene data were uploaded to an interactive viewer for visualisation and transparency (https://crclm2.serve.scilifelab.se/iss_panel_2.tmap).

Our spatial transcriptomic data aligned closely with known tissue biology [29, 30]. We observed that *ALB*, *CPS1*, and *C3* transcripts localised in hepatocyte-rich ‘liver’ region [29], *COL1A1* and *COL4A1* transcripts localised within stromal regions [29], and *CDX2*, *CTNNB1*, *KRT19*, and *EPCAM* transcripts were found within tumour pseudocrypts [5, 31, 32], consistent with their colorectal origin (Fig. 2d, e). We interpreted this spatial specificity as evidence supporting the validity of our ISS experiment. Moreover, we have demonstrated in a previous study strong concordance between ISS and immunohistochemistry for selected markers in CLM [24]. Additionally, studies benchmarking ISS against the Allen Brain Atlas have shown high spatial concordance, further supporting the reliability of ISS as a robust spatial transcriptomics method [33].

### Cell type composition across growth patterns revealed by unsupervised clustering

We began by applying unsupervised clustering to define cell populations based on molecular signatures. This yielded 12 clusters in Panel 1 and 15 clusters in Panel 2. We then computed the proportion of cells belonging to each cluster in each region. Supplementary Fig. 2A–D shows the regional distribution of clusters, together with marker genes for each cluster. Cluster annotations were based on marker genes (see Methods), prior literature, and spatial localisation (Supplementary Table 4). Panel 1 cluster annotations were given the suffix ‘−P1’ to distinguish them from Panel 2 clusters.

To ensure that clusterings were not driven by hyperparameter choices (*k*‑nearest neighbours, resolution, number of principal components [PCs]), we compared the selected parameters against a grid of alternatives and quantified concordance using normalised mutual information (NMI; Supplementary Fig. 3A). Cluster structure was stable across k and resolution, while increasing PCs primarily increased granularity rather than altering the major structure (Supplementary Fig. 3B).

We then compared regional cluster proportions between EHGP and RHGP samples using the Mann-Whitney U-test. Below, we describe the clusters that showed a significantly higher proportion of cells in specific regions of either EHGP or RHGP samples.

### RHGP is characterised by damaged hepatocytes

Clustering of the cells in Panel 2 revealed one cluster of hepatocytes (HC1), marked by *ALB* and *CPS1*, and three clusters with increased expression of *SAA1* and other inflammation-associated genes, including *CRP* and *PLA2G2A* ([17, 34–37], Fig. 3a). Following Wu et al. [17], who described *SAA1*⁺ hepatocytes at the tumour–liver interface as ‘damaged hepatocytes (DHC)’, we refer to these three clusters as DHC1-3.

Regular hepatocytes (HC1) predominated in the liver and liver front regions of EHGP samples, whereas damaged hepatocytes were more prevalent in RHGP samples (Fig. 3b). The cluster with the highest *SAA1* and *CRP* expression (DHC3) was almost exclusive to RHGP and represented 40% of liver-region cells (Supplementary Fig. 2a, b). In addition, cells belonging to these clusters were also detected at the tumour front of RHGP samples.

This result was robust across clustering parameter choices (Supplementary Fig. 3C). The mean read count of *SAA1*, *CRP*, and *PLA2G2A* across cells in the liver region was significantly higher in RHGP samples (Fig. 3c) than EHGP samples. Sample‑wise normalisation yielded similar results (Supplementary Fig. 4A), confirming that differences were not driven by overall sample‑specific tissue quality.

Spatial expression of these inflammation-associated genes was not confined to a few cell layers at the tumour-liver interface but extended several millimetres into the adjacent liver tissue (Fig. 3d). Immunohistochemical analysis of eleven RHGP samples validated our ISS findings, with high *SAA1* expression persisting millimetres into the liver orthogonal to the tumour (Fig. 3e and Supplementary Fig. 5).

Taken together, these findings define a distinct inflammation-associated zone in RHGP samples. In line with previous reports [17], this zone may contribute to a local microenvironment that influences tumour-host interactions, although functional consequences cannot be directly inferred from the present data.

### Bifunctional hepatocyte–cholangiocyte populations in RHGP samples

Clustering of Panel 2 cells also revealed a cholangiocyte-like cluster (CC1), characterised by expression of *SPP1* and *KRT7*, known cholangiocyte markers [38–40], see Fig. 3a. These cells were spatially localised within bile ducts outside the metastasis (Fig. 3f), supporting the cholangiocyte identity. These cholangiocyte-like cells were found in a higher proportion in the tumour region of RHGP samples compared with EHGP samples (Fig. 3g). The up regulation of these cells in the tumour region on RHGP was stable across clustering parameters (Supplementary Fig. 3C). Additionally, the mean read count of *SPP1* and *KRT7* per cell was higher in RHGP tumour regions compared to EHGP (Fig. 3h; Supplementary Fig. 4B). Figure 3i shows the spatial distribution of these cells, as well as of *SPP1* and *KRT7* in a RHGP metastasis near the tumour-liver interface. Interestingly, *CRP* and *C3* were also found in this cluster, known hepatocyte markers (see Fig. 3a), suggesting a potential bifunctional cellular state. To provide additional validation, multiplex immunofluorescence (COMET) staining confirmed the presence of SPP1⁺/KRT7⁺/CRP⁺ cells at RHGP interfaces, confirming cells positive for both cholangiocyte and hepatocyte markers, consistent with the transcriptional signature identified by ISS, see Supplementary Fig. 6.

Given the spatial arrangement and the known presence of co‑opted hepatocytes at RHGP interfaces in HE staining [5], these CC1 cells may represent co-opted hepatocytes that have adopted a cholangiocyte-associated transcriptional programme, consistent with a bifunctional hepatocyte–cholangiocyte phenotype (also known as progenitor-like cells). However, as this interpretation is based on marker expression and spatial context, definitive lineage assignment cannot be made. A plausible hypothesis is that damaged hepatocytes (*SAA1*⁺, *CRP*⁺, *PLA2G2A*⁺) surrounding the liver metastasis, upon co-option, undergo a transcriptional shift toward such a bifunctional phenotype [41].

### Neoplastic signatures in RHGP samples

The clustering of Panel 2 cells revealed four neoplastic cell clusters (NC1–3, NCγ), located in pseudocrypts of the tumour region and expressing established CLM markers including *KRT19*, *KRT18*, *EPCAM*, and *CDX2* (Supplementary Fig. 7A). Two clusters showed differential abundance between the growth patterns.

First, the NC3 cells were found in larger proportions on RHGP samples and marked by higher expression of *KRT18*, *GSN*, and *SLPI* (Supplementary Fig. 7A, B). Mean read count comparison confirmed a modest increase in *KRT18* expression in the RHGP tumour region, while *GSN* and *SLPI* did not differ significantly (Supplementary Fig. 7C). We therefore consider NC3 to be largely driven by slightly elevated *KRT18*, but given the low gene count detected, we remain tentative in interpreting this cluster.

Second, NCγ cells were enriched in EHGP samples and marked by genes associated with interferon gamma signalling, consistent with the broader IFNγ-related signature described below.

### Interferon-γ signalling in EHGP samples

We identified two interferon-γ (IFNγ)-induced clusters (NCγ and CAFγ), marked by high expression of hallmark IFN-induced genes (*CXCL10*, *CXCL11*, *IFIT3*, *GBP4*; refs. [42–44]; Fig. 4a). These clusters were more abundant in EHGP samples in the tumour and stroma regions (Fig. 4b).

We directly compared the mean read count of the IFNγ-induced genes and found higher levels in stromal, tumour, and tumour front regions of EHGP samples than in RHGP samples (Fig. 4c and Supplementary Fig. 4D).

Neighbourhood enrichment analysis (methods) with neighbourhoods defined by *k* = 5, 15, and 30 nearest neighbours revealed that CAFγ cells frequently neighboured NCγ cells and co‑localised with an immune population (MΦ) across stroma, portal tissue, and tumour regions (Fig. 4d).

These IFNγ-induced cells localised primarily at the tumour-stroma interface (Fig. 4e), with CAFγ concentrated in the capsule and occasionally infiltrating the tumour stroma, and NCγ primarily within the tumour of EHGP samples. Together, these findings suggest that IFNγ signalling may shape the immune microenvironment of EHGP samples.

### Cytotoxic T-cells enriched in the capsule of EHGP samples

While examining the data, we observed increased *GZMA* reads in EHGP samples, a finding also reported in bulk RNA‑seq analyses of HGPs by Latacz et al. [20]. To identify the cell type expressing *GZMA*, we performed neighbourhood‑enrichment tests (radii 5, 10, 20 µm) around *GZMA* reads. Genes with the highest enrichment around *GZMA* reads included *CXCR4*, *CD8A*, and *CD74* (Fig. 5a), consistent with cytotoxic T-cells [20, 45]. We then labelled all *GZMA*^+^ cells as cytotoxic cells (CTX) and compared their proportions between the growth patterns. A larger proportion was found on EHGP samples (Fig. 5b). When comparing CTX cells relative to total immune cells (CTX, MΦ), EHGP samples still showed a higher proportion (Fig. 5c).

Additionally, the mean read count across cells in the stroma region of the T-cell-related genes *CD8A, GZMA, CXCR4*, and *CD74* was higher in EHGP samples compared to RHGP (Fig. 5d and Supplementary Fig. 4E).

To further explore spatial interactions, we performed a neighbourhood enrichment analysis (using *k* = 5, 15, and 30 nearest neighbours to define neighbourhoods) in combined stroma and portal regions. This analysis showed that CTX cells were frequently located near interferon–γ–induced CAFs (CAFγ), macrophages (MΦ), and activated hepatic stellate cells (aHSC1; Fig. 5e). *CXCL12* expression in aHSC1 cells (Supplementary Fig. 8), together with *CXCR4* expression in CTX cells, is consistent with a potential role for the *CXCR4*–*CXCL12* axis in the spatial organisation of cytotoxic T-cells within the capsule.

These findings point to a structured microenvironment in EHGP samples in which CTX cells are spatially associated with specific stromal populations. To explore the broader organisation of the capsule microenvironment, we next examined the zonation of the fibrotic capsule along the tumour-capsule-liver axis.

### Molecular zonation of the fibrotic capsule reflects wound healing, immune signalling and vascular remodelling

#### Defining capsule zonation in EHGP samples

Moro et al. and we [19, 24] have shown that the fibrotic capsule surrounding encapsulated histopathological growth pattern (EHGP) samples is spatially zonated. This zonation appears to reflect a liver injury response rather than tumour-induced desmoplasia.

**Fig. 6:**
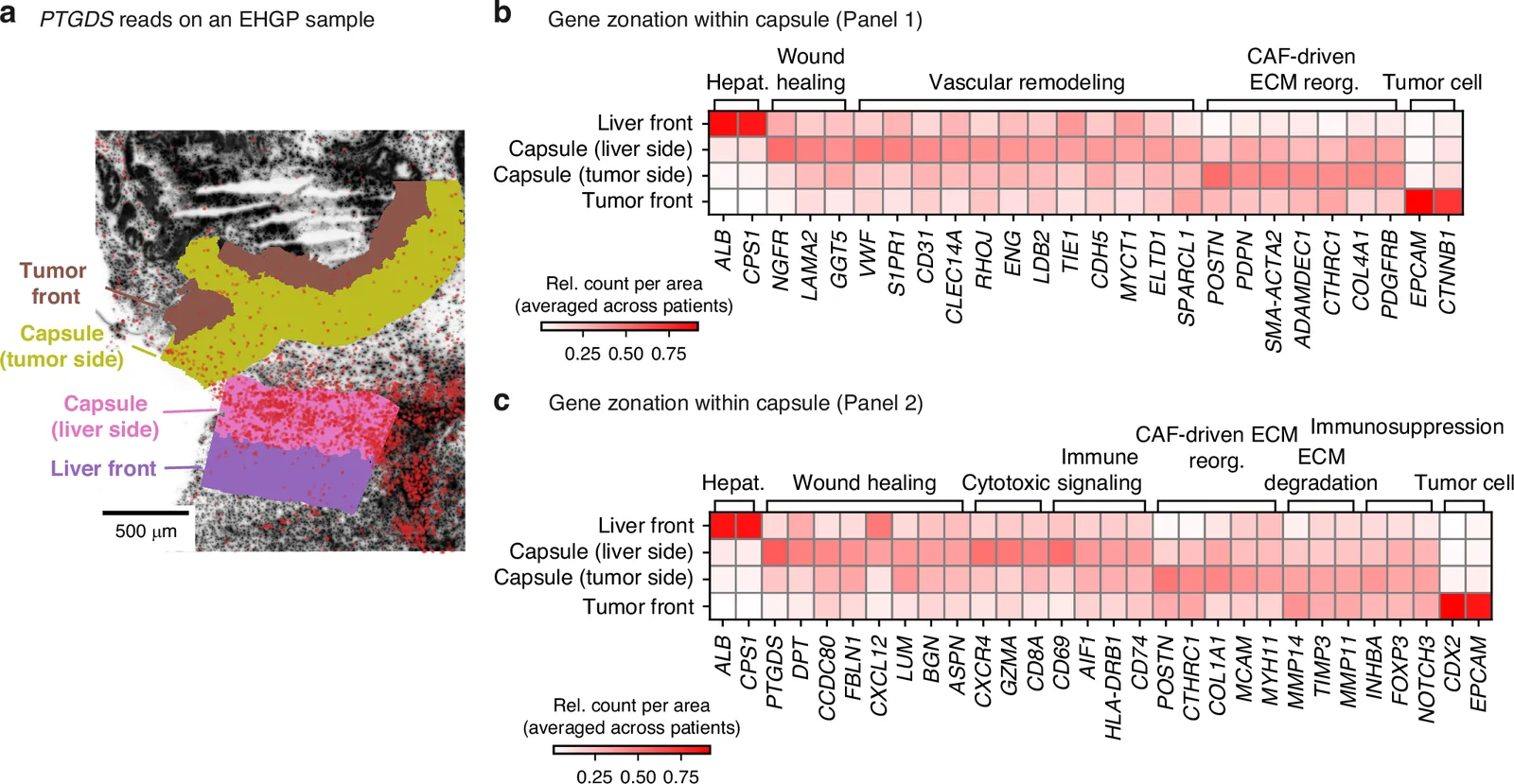
Spatial gene expression analysis reveals the zonation of the capsule. **a** The read count per area of an example gene, *PTGDS, is* computed in four different regions: liver front, capsule (liver side), capsule (tumour side), and tumour front. The values are normalised by their total sum across regions, resulting in a relative count. The average (across patients) relative count is shown for a subset of genes in Panel 1 (**b**) and Panel 2 (**c**) as heatmaps.

To further explore these spatial patterns, we defined four compartments within EHGP-labelled samples: capsule (liver side), capsule (tumour side), liver front, and tumour front (Fig. 6a). These regions were used to analyse the molecular patterns across the tumour-capsule-liver axis.

For each region and gene, read counts were normalised by the total counts across all four regions, producing a relative count for each region. This was performed across all EHGP samples and genes. The average relative count (across EHGP samples) for selected genes is shown in Fig. 6b, c, with all region-associated genes listed in Supplementary Fig. 9a, b.

#### Liver-side of the capsule: Hepatic wound healing response

On the liver side of the capsule, we observed signatures of HSC (Fig. 6b, c). The expression of genes such as *NGFR*, *LAMA2*, *GGT5*, *CXCL12*, *PTGDS*, *DPT*, *FBLN1*, *CCDC80*, *BGN*, and *ASPN* [40] supports the presence of activated HSCs in this region. The two associated HSC clusters, aHSC1 and aHSC2-P1, coexpressed several of these genes (Supplementary Fig. 8), and were likewise localised in the liver side of the capsule (Supplementary Fig. 9B).

These findings are consistent with a regenerative liver injury response rather than tumour-driven desmoplasia. Notably, the chemokine *CXCL12* was expressed by aHSC1 cells (Supplementary Fig. 8), consistent with Payen et al. [40], where they performed single-cell sequencing to uncover the heterogeneity of HSCs.

#### Liver-side of the capsule: Cytotoxic T-cells and immune signalling

Immune signalling was identified at the liver side of the capsule, with expression of genes including *CD8A, GZMA, CXCR4, CD69, HLA-DRB1*, and *CD74* (Fig. 6c), implying the presence of cytotoxic T-cells (*CD8A, GZMA, CXCR4, CD74*) [20, 45] and antigen-presenting cells (*CD69, HLA-DRB1*). The spatial co-localisation of *CXCR4*⁺ cytotoxic T-cells and *CXCL12*⁺ HSCs at the liver side of the capsule is compatible with a potential role for the *CXCR4-CXCL12* axis in cytotoxic T-cell localisation in this region [46, 47], a hypothesis that needs further testing.

#### Liver-side of the capsule: vascular remodelling and endothelial activation

The liver side of the capsule showed signs of vascular remodelling. Expression of angiogenesis- and endothelium-associated genes [48], including *VWF*, *S1PR1*, *CLEC14A*, *TIE1*, *RHOJ*, *MYCT1*, *ELTD1*, *ENG*, *LDB2*, *CDH5*, and *SPARCL1*, was enriched in this region (Fig. 6b). Additionally, the endothelial marker *CD31* was detected, suggesting active remodelling of the vasculature, consistent with a tissue regeneration environment.

#### Tumour-side of the capsule: ECM remodelling and immunosuppression

The tumour side of the capsule displayed a distinct set of biological programmes. Genes associated with extracellular matrix (ECM) production and CAFs were enriched, including *POSTN*, *PDPN*, *ACTA2* (*SMA*), *ADAMDEC1*, *CTHRC1*, *COL1A1*, *COL4A1*, *MCAM*, *MYH11*, and *PDGFRB*, see Fig. 6b, c [24, 49]. ECM turnover was indicated by the presence of metalloproteases *MMP11*, *MMP14*, and their inhibitor *TIMP3*, suggesting active-matrix remodelling that may support tumour progression, see Fig. 6c [50, 51]. Additionally, we noted expression of *NOTCH3*, *INHBA*, and *FOXP3*, potentially indicating an immunosuppressive microenvironment adjacent to the tumour [52–54].

## Discussion

We identified three populations of damaged hepatocytes in Panel 2 (DHC1-3) located in the liver and liver front regions (Fig. 3a, b). These cells were characterised by expression of hepatocyte inflammation-associated genes (*SAA1, CRP, C3, PLA2G2A*; Fig. 3a). DHC1 cells expressed these markers at low levels, whereas DHC2 represented a rare subset (~2% of hepatocytes) additionally expressing IFNγ-induced genes (*GBP4, CXCL11*). In contrast, DHC3 showed high hepatocyte-inflammation-associated gene expression and was enriched in the liver and liver front of RHGP-labelled lesions (Supplementary Fig. 2A–D), which are associated with a worse clinical course compared with EHGP. Conversely, the HC1 population lacked hepatocyte-injury gene expression and was more abundant in EHGP samples in the liver and liver front (Fig. 3B), which are linked to more favourable outcomes [20].

These spatial and transcriptional differences in hepatocyte populations may reflect the direct interaction between tumour cells and hepatocytes in RHGP, potentially driving a localised hepatocyte-driven inflammatory response. Further support for this is a recent study by Wu et al. [17], who demonstrated that a population of *SAA1*^*+*^ hepatocytes is present at the tumour-hepatocyte interface in primary liver cancer, where they participate in modulating the tumour microenvironment. They showed that *SAA1*^*+*^ hepatocytes secrete SAA proteins, which act as inflammatory mediators that recruit macrophages and promote their M2-like polarisation, establishing a local immunosuppression and thus, tumour progression and metastasis [17].

Furthermore, in RHGP samples, we observed that damaged hepatocytes (DHC1, DHC3) were present in the tumour front region. The RHGP is characterised by preserving the architecture of the liver and hepatocytes are being replaced by tumour cells. These damaged hepatocytes likely represent co-opted hepatocytes that are actively reprogrammed by tumour-derived signals to support cancer cell survival, proliferation, invasion, and metastasis [8].

In RHGP samples, we identified a population of cells at the tumour-liver interface expressing cholangiocyte- and hepatocyte-associated markers (*SPP1*⁺, *KRT7*⁺, CRP^+^), consistent with a co-opted bifunctional hepatocyte-cholangiocyte-like transcriptional state (Fig. 3f–i). This was also validated by multiplex IHC (Supplementary Fig. 6). Both this cell population and the adjacent damaged hepatocytes have been described in settings of chronic liver injury and may also arise in response to the presence of a growing metastasis [41, 55]. Such a progenitor-like state has been proposed to reflect regenerative responses of the liver to tissue stress. We speculate that this regenerative niche could be permissive or supportive for neoplastic cells in RHGP, which owing to their less differentiated state, may be particularly responsive to these cues [56].

In the tumour region of RHGP samples, we observed an enrichment of NC3, a neoplastic cell cluster marked by elevated *KRT18* expression (Supplementary Fig. 6A, B). This marker has previously been linked to poor prognosis and tumour invasion in CRC [57]. However, the increase in *KRT18* expression was modest, though significant, and the limited gene counts per cell in ISS make absolute quantification uncertain. We therefore present this as an observation that requires further validation, rather than a definitive RHGP-specific feature.

In our previous work [24], we described zonation within the fibrotic capsule of EHGP samples using Panel 1. We observed that the tumour-facing side of the capsule expressed markers such as *POSTN*, consistent with stromal remodelling influenced by the tumour. By contrast, the liver-facing side expressed *NGFR*, suggesting a stromal state resembling a non-specific wound healing response. This is in line with results reported by Moro et al. [19].

Consistent with this injury‑response framework, we also observed damaged (*CRP*^+^) hepatocytes not only in RHGP samples but also in the liver and liver‑front regions of EHGPs. This mirrors the *CRP*‑expressing hepatocytes described by Moro et al. [19] adjacent to encapsulated metastases, reflecting hepatocyte stress as part of a generic liver‑injury programme that engages stromal compartments, including HSCs [58].

Building on this framework, the present study identifies additional genes (*PTGDS*, *DPT*, *CCDC80*, *IGFBP3, FBLN1*, *CXCL12)* that were enriched on the liver-facing side of the capsule (Fig. 6c). These genes are associated with HSCs [40] and localise to both portal tracts and the capsule-liver interface (Supplementary Fig. 2A—D and Fig. 6a–c). Our unsupervised clustering also indicated that these genes were expressed by the aHSC1 cluster of cells (Supplementary Fig. 8). This expression pattern further reinforces the idea that the fibrotic capsule arises as a liver-driven, injury-like response, rather than being induced by tumour-derived desmoplasia, and supports a model in which the capsule originates from local activation of HSCs and recapitulates features of tissue repair and regeneration.

While the liver‑facing side of the capsule contained *CXCL12*⁺ HSCs, we also noted *CXCR4*⁺ cytotoxic T‑cells positioned in the same outer region (Fig. 6c and Supplementary Fig. 9B). Hypothetically, the HSCs may play a role in recruiting and retaining cytotoxic T-cells to this niche via *CXCL12*—*CXCR4* signalling, although this mechanism requires further experimental validation. Notably, the abundance of cytotoxic T-cells was elevated in EHGP samples (Fig. 5b—d), which aligns with previous studies reporting increased *GZMA* signalling and a higher frequency of *CD8*^+^ T-cells in these lesions [20, 59].

Cytotoxic T-cells are well-established sources of interferon gamma [42]. Spatial analysis revealed a spatial association between cytotoxic T-cells and IFNγ-responsive cell types-including CAFγ cells expressing *CXCL10*, *CXCL11*, *GBP4*, and *IFIT3*-consistent with the role of cytotoxic T-cell-derived IFNγ in stimulating these neighbouring cell populations (Fig. 5e).

Additionally, the chemokines produced by IFNγ-stimulated CAFγ cells, such as *CXCL10* and *CXCL11*, may attract *CXCR3*^+^ monocytes and macrophages into the tumour stroma [60]. This recruitment leads to local accumulation of macrophages around CAFγ cells, contributing to increased immune cell infiltration, an observation consistent with the EHGP growth pattern [61]. Nonetheless, further experimental validation is warranted to substantiate these findings.

In addition to immune cell interactions, CAFγ cells are found adjacent to a distinct neoplastic cell subtype, NCγ (Fig. 4d), characterised by the expression of IFN-associated genes such as *CXCL10*, *CXCL11*, *IFIT3*, and *GBP4*. Previous research demonstrates that IFN-sensitive neoplastic cells in colorectal cancer can respond to local IFN signalling by undergoing increased apoptosis and reduced proliferation [62]. This mechanism may help explain the more favourable prognosis observed in patients with EHGP lesions [5], although further studies are required to corroborate this hypothesis.

In conclusion, our spatial transcriptomic analysis reveals distinct molecular and cellular features underpinning encapsulated and replacement HGPs in CLM. We demonstrated that RHGP samples are characterised by damaged hepatocyte populations (e.g. DHC3), specific *KRT18*^+^ neoplastic cells and bifunctional hepatocyte-cholangiocyte cells that favour tumour integration. By contrast, EHGP samples are associated with interferon-γ-driven signalling in CAFγ and NCγ populations, formation of a localised fibrotic capsule, and recruitment of cytotoxic immune cells. These features, in EHGP, support a dynamic stromal and immune landscape that may restrain tumour invasion while promoting structural re-organisation. The EHGP exists in ontologically naïve patients with CLM, but the degree of EHGP increases in patients given oncological therapy [19]. This demonstrates that patients with RHGP CLM could likely be induced to a more favourable HGP by oncological treatment. It is therefore crucial to understand the mechanisms of the HGP and if this can be used to improve outcomes for CLM patients.

This study is, to our knowledge, the first study focusing on the differences in the HGP in patients with CLM by targeted ISS approach. We feel that the limitations of our study should be discussed. An important limitation of this study is the dependence of the findings on the selected ISS gene panels. Both panels were intentionally designed to address specific biological questions informed by our previous work, with a strong emphasis on stromal biology, particularly HSCs and CAFs. Consequently, these panels provide high resolution for stromal states and fibroblast heterogeneity but offer more limited coverage of other potentially relevant compartments.

Several immune lineages and functional states are under-represented, including diverse T-cell activation and exhaustion programmes, myeloid and macrophage heterogeneity, and immunosuppressive pathways beyond interferon signalling. Therefore, the absence of differential expression or spatial differences for these processes should not be interpreted as evidence of biological equivalence between replacement and encapsulated growth patterns, but rather as a reflection of limited panel coverage.

While the expanded CAF-focused panel enabled the identification of additional stromal populations, such as CAFγ and damaged hepatocytes, broader immune and tumour-intrinsic programmes may not be fully captured. Accordingly, conclusions regarding RHGP/EHGP biology should be interpreted within the context of the pathways and cell types targeted by the panels, and future studies using larger cohorts and more comprehensive or unbiased transcriptomic approaches will be required to fully resolve immune and tumour ecosystem differences between growth patterns.

We do, however, believe that our findings offer a deeper understanding of the mechanisms driving encapsulation and suggest potential targets to explore, potentially promoting EHGP development and improving outcomes for patients with CLM. Taken together, these insights not only refine our understanding of CLM heterogeneity but may inform future efforts for further research to stratify patients and potentially tailor therapeutic interventions based on the hepatic growth patterns.

## Supplementary information

Supplementary Material

Supplementary Table S2

## Data Availability

To ensure open and easy access, the data, including image data (nuclear stain, HE), genes-per-cell matrices, and raw sequencing reads, have been made available through an online viewer, where they can be visualised and downloaded: https://crclm2.serve.scilifelab.se/iss_panel_2.tmap. The raw ISS image data is over 50TB in size and available on request.

## Code availability

Python code for analysis and reproducing figures is available at https://github.com/axanderssonumu/iss_crclm.
