# Supplementary material for: Spatial transcriptomics differences of histopathological growth patterns in colorectal cancer liver metastases
Source: Br J Cancer. 2026 Jul 28;135(9):1499–511. doi: 10.1038/s41416-026-03567-y (PMC13675213; doi:10.1038/s41416-026-03567-y)
Supplement: Supplementary file 1 [file 41416_2026_3567_MOESM1_ESM.pdf]

Supplementary Tables

Supplemental Table S1: Cohort overview. The dataset comprises 26 samples originating from 19 patients. When multiple samples share the same Patient ID, they represent distinct regions of interest sampled from the same metastasis. Histopathological growth patterns (HGPs) are annotated as EHGP (encapsulating HGP) and RHGP (replacement HGP). Checkmarks (✓) indicate inclusion in the corresponding analysis panel; crosses (✗) indicate exclusion due to poor tissue and staining quality. When two samples from the same patient shared the same HGP (marked by *), their measurements were averaged before statistical testing to prevent inflation of independent observations.

Sample ID	Patient ID	Used in Panel 1	Used in Panel 2	Growth pattern
ENC-P01-S01	P01 *	✓	✓	EHGP
ENC-P01-S02	P01 *	✓	✓	EHGP
ENC-P02-S03	P02	✓	✓	EHGP
ENC-P03-S04	P03	✓	✓	EHGP
REP-P03-S14	P03	✓	✓	RHGP
ENC-P04-S05	P04	✓	✓	EHGP
ENC-P05-S06	P05	✓	✓	EHGP
REP-P05-S16	P05	✓	✓	RHGP
ENC-P06-S07	P06	✓	✓	EHGP
ENC-P07-S08	P07	✓	✓	EHGP
ENC-P08-S09	P08	✓	✓	EHGP
ENC-P10-S11	P10	✓	✓	EHGP
REP-P11-S12	P11 *	✓	✓	RHGP
REP-P11-S13	P11 *	✓	✗	RHGP
REP-P12-S15	P12	✓	✓	RHGP
REP-P13-S17	P13	✓	✓	RHGP
REP-P14-S18	P14 *	✓	✓	RHGP
REP-P14-S19	P14 *	✓	✓	RHGP
REP-P15-S20	P15	✓	✓	RHGP
REP-P16-S21	P16	✗	✓	RHGP
REP-P16-S27	P16	✓	✗	RHGP
REP-P17-S22	P17 *	✓	✓	RHGP
REP-P17-S23	P17 *	✓	✓	RHGP
REP-P18-S24	P18	✓	✓	RHGP
REP-P19-S25	P19	✓	✗	RHGP
REP-P20-S26	P20	✓	✗	RHGP

Supplementary Table S2: See attached EXCEL-document

Supplemental Table S3: Antibody and COMET details.

Name	Target	Lot	Clone	Host	Manufacturer	Catalog Number	Dilution factor	Incubation time (min)	Acquisition channel
Hepatocyte (Concentrate) HSA HepPar	HSA	41597648	OCH1E5	mouse	Agilent	M715801-2	1 : 300	4	Cy5
CDX2 (D11D10) Rabbit mAb	CDX2	5	D11D10	rabbit	Cell Signaling Technology	12306S	1 : 50	4	Cy5
Cytokeratin 7 (KRT7)	KRT7	41653921	OV-TL 12/30	mouse	Agilent	M701801-2	1 : 200	8	TRITC
Anti-Osteopontin antibody (SPP1)	SPP1	1089668-27	EPR21139-316	rabbit	Abcam	Ab214050	1 : 800	4	Cy5
Anti-C Reactive Protein antibody CRP	CRP	1016267NA26	Y284	rabbit	Abcam	AB32412	1 : 1500	4	TRITC

Supplementary Table S4: Annotation of clusters from Panels 1 and 2 according to existing bibliography and spatial location.

**Annotation Panel 1**	**Marker genes**	**Rationale**
VASC-P1	*COL4A1, CD93, IGFBP7, ESAM, MCAM, THBD, CLEC14A*	By their spatial location, and the following bibliography, we annotate this cluster as vascular cells (VASC-P1)(1).
CAF4-P1	*COL4A1, IGFBP7, POSTN, SMA-ACTA2, CTHRC1, PDGFRB*	By their spatial location; and the following bibliography, we annotate this cluster as cancer-associated fibroblast type 4 (CAF4-P1) (2,3).
aHSC2-P1	*COL4A1, IGFBP7, PDGFRB, LAMA2, ENG, NGFR, GGT5*	By their spatial location; and the following bibliography, we annotate this cluster as activated hepatic stellate cell type 2 (aHSC2-P1) (2,4).
CC2-P1	*SPP1*	By their spatial location; and the following bibliography, we annotate this cluster as cholangiocyte type 2 (CC2-P1) (5).
STROMAL1-P1	*MCAM, MXD4, LDB2, MAX, FZD9, ROBO4*	By their spatial location; and the information from the Human protein Atlas, we annotate this cluster as STROMAL1-P1 (6–11).
STROMAL2-P1	*MCAM, RSPO1, CDH5, SPARCL1, NOC4L, CD45*	By their spatial location; and the information from the Human protein Atlas , we annotate this cluster as STROMAL2-P1 (11–16)
HC2-P1	*CPS1, ALB*	By their strong presence in the Liver and Liver front regions; and the information from the Human protein Atlas , we annotate this cluster as CPS1 high hepatocytes type 2 (HC2-P1) (17,18).
HC3-P1	*CPS1, ALB, GPX3*	By their strong presence in the Liver and Liver front regions; and the information from the Human protein Atlas , we annotate this cluster as hepatocytes type 3 (HC3-P1) (17–19).
HC4-P1	*CPS1, ALB*	By their strong presence in the Liver and Liver front regions; and the information from the Human protein Atlas , we annotate this cluster as hepatocyte type 4 (HC4-P1) (17,18).
NC4-P1	*WNT11, L1CAM, RHOA, PRKCZ, DVL1, MSLN*	By their strong presence in the tumor pseudocrypts in the Tumor and Tumor front regions, and the following bibliography, we annotate this cluster as neoplastic cell 4 (NC4-P1) (20).
NC5-P1	*AXIN2, PTCH1, CD29, CDH1, EPCAM, ARPC3*	By their strong presence in the tumor pseudocrypts in the Tumor and Tumor front regions, and the following bibliography, we annotate this cluster as as neoplastic cell 5 (NC5-P1) (20).
NC6-P1	*DVL1, AXIN2, CDH1, SLC19A1, CALM3, CAMK2G, PTK2, MLXIP, CTNNB1*	By their strong presence in the Tumor and Tumor front regions; and the following bibliography, we annotate this cluster as neoplastic cell 6 (NC6-P1) (20).
**Annotation Panel 2**	**Marker genes**	**Rationale**
aHSC1	*CXCL12, IGFBP3, BGN, FOSB, PTGDS*	By their strong presence in the portal tissue region and the following bibliography, we annotate this cluster as activated hepatic stellate cells type 1 (aHSC1) (4).

CAF1	*BGN, SPARC, ACTA2, VIM*	By their strong presence in the stroma region; and the following bibliography, we annotate this cluster as cancer associated fibroblast type 1 (CAF1) (3,23,24).
CAF2	*THBS2, PDGFRB, COL1A1, COL4A1, POSTN, FN1, COL3A1, LTBP2*	By their strong presence in the Stroma region; and the following bibliography, we annotate this cluster as cancer associated fibroblast type 2 (CAF2) (3,24,25).
CAFγ	*CXCL10, IFIT3, GBP4*	This cell population express the same genes as the CAFs described above the IFNγ^{+} associated genes (*IFIT3, GBP4* and *CXCL10*).Thus, we annotate this cluster as CAFγ(26–29). Strong presence in stroma.
NCγ	*CXCL11, CXCL10, IFIT3, GBP4, SOX9, LDHA, ITGB1, PLA2G2A*	This cell population express the same genes as the NC1 described in this table plus the IFNγ^{+} associated genes (*IFIT3, GBP4, CXCL10,* and *CXCL11*) genes Thus, we annotate this cluster as neoplastic cell IFNγ (NCγ) (20,27–32). Strong presence in Tumor and Tumor front regions.
CAF3	*SFRP4, FCGR3A, PDGFRA, MMP11*	By their strong presence in the Stroma region; and the following bibliography, we annotate this cluster as cancer associated fibroblast type 3 (CAF3) (24,33,34)
CC1	*KRT7, SPP1*	By their spatial location; and the following bibliography, we annotate this cluster as cholangiocyte type 1 (CC1) (5,35).
MΦ	*VIM, CXCR4, CD74, HLA-DRB1, CD163*	By the following bibliography, we annotate this cluster as macrophages (MΦ) (36–38).
NC1	*ACTR2, KRT19, CD44, HNF1A, KRT20, EPCAM, CDX2, LDHA, ITGB1, SOX9*	By their strong presence in the tumor pseudocrypts at the Tumor and Tumor front regions and the following bibliography, we annotate this cluster as neoplastic cell type 1 (NC1) (20).
NC3	*KRT19, KRT20, CDX2, KRT18, SLPI, ASCL2*	By their strong presence in the tumor pseudocrypts in the Tumor and Tumor front regions; and the following bibliography, we annotate this cluster as neoplastic cell type 3 (NC3) (20).
NC2	*KRT19, CDX2, ASCL2, AXIN2, PROM1, HES1, EPHB3, FOXA2*	By their strong presence in the tumor pseudocrypts in the Tumor and Tumor front regions; and the following bibliography, we annotate this cluster as neoplastic cell type 2 (NC2)(20).
HC1	*MT2A, ARG1, ERRFI1, ALB, CPS1, TF, C3*	By their strong presence in the Liver and Liver front regions; and the information from the Human protein Atlas , we annotate this cluster as hepatocyte type 1 (HC1) (17,18,39–43).
DHC1	*MT2A, ARG1, ALB, C3, CRP*	By their strong presence in the Liver and Liver front regions and the information from the Human protein Atlas , we annotate this cluster as damaged hepatocytes type 1 (DHC1) (17,39,40,42,44,45).
DHC2	*MT2A, ARG1, ALB, C3, CRP, SAA1, CXCL11, GBP4*	By their strong presence in the Liver and Liver front regions; and the information from the Human protein Atlas , we annotate this cluster as damaged hepatocytes type 2 (DHC2) (17,39,40,42,44,45).
DHC3	*MT2A, C3, CRP, SAA1, PLA2G2A*	By their strong presence in the Liver and Liver front regions; and the information from the Human protein Atlas , we annotate this cluster as damaged hepatocytes type 3 (DHC3) (39,42,44–46).

References

1. Masiero M, Simões FC, Han HD, Snell C, Peterkin T, Bridges E, et al. A Core Human Primary Tumor Angiogenesis Signature Identifies the Endothelial Orphan Receptor ELTD1 as a Key Regulator of Angiogenesis. Cancer Cell. 2013 Aug;24(2):229–41.

2. Andersson A, Escriva Conde M, Surova O, Vermeulen P, Wählby C, Nilsson M, et al. Spatial Transcriptome Mapping of the Desmoplastic Growth Pattern of Colorectal Liver Metastases by In Situ Sequencing Reveals a Biologically Relevant Zonation of the Desmoplastic Rim. Clinical Cancer Research. 2024 Oct 1;30(19):4517–29.

3. Giguelay A, Turtoi E, Khelaf L, Tosato G, Dadi I, Chastel T, et al. The landscape of cancer-associated fibroblasts in colorectal cancer liver metastases. Theranostics. 2022 Oct 31;12(17):7624–39.

4. Payen VL, Lavergne A, Alevra Sarika N, Colonval M, Karim L, Deckers M, et al. Single-cell RNA sequencing of human liver reveals hepatic stellate cell heterogeneity. JHEP Reports. 2021 Jun 1;3(3):100278.

5. Song Z, Chen W, Athavale D, Ge X, Desert R, Das S, et al. Osteopontin Takes Center Stage in Chronic Liver Disease. Hepatology. 2021 Apr;73(4):1594.

6. MXD4 protein expression summary - The Human Protein Atlas [Internet]. [cited 2025 May 8]. Available from: https://www.proteinatlas.org/ENSG00000123933-MXD4

7. LDB2 protein expression summary - The Human Protein Atlas [Internet]. [cited 2025 May 8]. Available from: https://www.proteinatlas.org/ENSG00000169744-LDB2

8. MAX protein expression summary - The Human Protein Atlas [Internet]. [cited 2025 May 8]. Available from: https://www.proteinatlas.org/ENSG00000125952-MAX

9. FZD9 protein expression summary - The Human Protein Atlas [Internet]. [cited 2025 May 8]. Available from: https://www.proteinatlas.org/ENSG00000188763-FZD9

10. ROBO4 protein expression summary - The Human Protein Atlas [Internet]. [cited 2025 May 8]. Available from: https://www.proteinatlas.org/ENSG00000154133-ROBO4

11. MCAM protein expression summary - The Human Protein Atlas [Internet]. [cited 2025 May 8]. Available from: https://www.proteinatlas.org/ENSG00000076706-MCAM

12. RSPO1 protein expression summary - The Human Protein Atlas [Internet]. [cited 2025 May 8]. Available from: https://www.proteinatlas.org/ENSG00000169218-RSPO1

13. CDH5 protein expression summary - The Human Protein Atlas [Internet]. [cited 2025 May 8]. Available from: https://www.proteinatlas.org/ENSG00000179776-CDH5

14. SPARCL1 protein expression summary - The Human Protein Atlas [Internet]. [cited 2025 May 8]. Available from: https://www.proteinatlas.org/ENSG00000152583-SPARCL1

15. NOC4L protein expression summary - The Human Protein Atlas [Internet]. [cited 2025 May 8]. Available from: https://www.proteinatlas.org/ENSG00000184967-NOC4L

16. PTPRC protein expression summary - The Human Protein Atlas [Internet]. [cited 2025 May 8]. Available from: https://www.proteinatlas.org/ENSG00000081237-PTPRC

17. ALB protein expression summary - The Human Protein Atlas [Internet]. [cited 2025 May 8]. Available from: https://www.proteinatlas.org/ENSG00000163631-ALB

18. CPS1 protein expression summary - The Human Protein Atlas [Internet]. [cited 2025 May 8]. Available from: https://www.proteinatlas.org/ENSG00000021826-CPS1

19. GPX3 protein expression summary - The Human Protein Atlas [Internet]. [cited 2025 May 8]. Available from: https://www.proteinatlas.org/ENSG00000211445-GPX3

20. Chen B, Scurrah CR, McKinley ET, Simmons AJ, Ramirez-Solano MA, Zhu X, et al. Differential pre-malignant programs and microenvironment chart distinct paths to malignancy in human colorectal polyps. Cell. 2021 Dec 22;184(26):6262-6280.e26.

23. Gao Y, Yin SP, Xie XS, Xu DD, Du WD. The relationship between stromal cell derived SPARC in human gastric cancer tissue and its clinicopathologic significance. Oncotarget. 2017 Sep 21;8(49):86240–52.

24. Han C, Liu T, Yin R. Biomarkers for cancer-associated fibroblasts. Biomarker Research. 2020 Nov 11;8(1):64.

25. Li J, Tang Y, Long F, Tian L, Tang A, Ding L, et al. Integrating bulk RNA-seq and scRNA-seq analyses revealed the function and clinical value of thrombospondins in colon cancer. Computational and Structural Biotechnology Journal. 2024 Dec 1;23:2251–66.

26. Koppensteiner L, Mathieson L, Neilson L, O'Connor RA, Akram AR. IFNγ and TNFα drive an inflammatory secretion profile in cancer-associated fibroblasts from human non-small cell lung cancer. FEBS Lett. 2025 Mar;599(5):713–23.

27. Du W, Frankel TL, Green M, Zou W. IFNγ signaling integrity in colorectal cancer immunity and immunotherapy. Cell Mol Immunol. 2022 Jan;19(1):23–32.

28. Chai B, Li Y, Guo Y, Zhang Z, Jia K, Chai X, et al. ETV7 promotes colorectal cancer progression through upregulation of IFIT3. Funct Integr Genomics. 2024 Jan 10;24(1):8.

29. Tretina K, Park ES, Maminska A, MacMicking JD. Interferon-induced guanylate-binding proteins: Guardians of host defense in health and disease. J Exp Med. 2019 Mar 4;216(3):482–500.

30. Su C, Mo J, Dong S, Liao Z, Zhang B, Zhu P. Integrinβ-1 in disorders and cancers: molecular mechanisms and therapeutic targets. Cell Communication and Signaling. 2024 Jan 26;22(1):71.

31. Liang X, Duronio GN, Yang Y, Bala P, Hebbar P, Spisak S, et al. An Enhancer-Driven Stem Cell–Like Program Mediated by SOX9 Blocks Intestinal Differentiation in Colorectal Cancer. Gastroenterology. 2022 Jan 1;162(1):209–22.

32. Wang Y, Nie H, Liao Z, He X, Xu Z, Zhou J, et al. Expression and Clinical Significance of Lactate Dehydrogenase A in Colon Adenocarcinoma. Front Oncol [Internet]. 2021 Jul 9 [cited 2025 May 8];11. Available from: https://www.frontiersin.orghttps://www.frontiersin.org/journals/oncology/articles/10.3389/fonc.2021.700795/full

33. Ning L, Quan C, Wang Y, Wu Z, Yuan P, Xie N. scRNA-seq characterizing the heterogeneity of fibroblasts in breast cancer reveals a novel subtype SFRP4+ CAF that inhibits migration and predicts prognosis. Front Oncol [Internet]. 2024 Apr 15 [cited 2025 May 8];14. Available from: https://www.frontiersin.orghttps://www.frontiersin.org/journals/oncology/articles/10.3389/fonc.2024.1348299/full

34. Eiro N, Cid S, Fernández B, Fraile M, Cernea A, Sánchez R, et al. MMP11 expression in intratumoral inflammatory cells in breast cancer. Histopathology. 2019;75(6):916–30.

35. Wang Z, Faria J, van der Laan LJW, Penning LC, Masereeuw R, Spee B. Human Cholangiocytes Form a Polarized and Functional Bile Duct on Hollow Fiber Membranes. Front Bioeng Biotechnol [Internet]. 2022 Jun 24 [cited 2025 May 8];10. Available from:

https://www.frontiersin.orghttps://www.frontiersin.org/journals/bioengineering-and-biotechnology/articles/10.3389/fbioe.2022.868857/full

36. Li Z, Wu T, Zheng B, Chen L. Individualized precision treatment: Targeting TAM in HCC. Cancer Letters. 2019 Aug 28;458:86–91.

37. Li RQ, Yan L, Zhang L, Zhao Y, Lian J. CD74 as a prognostic and M1 macrophage infiltration marker in a comprehensive pan-cancer analysis. Sci Rep. 2024 Apr 7;14(1):8125.

38. Zhang Y, Zhang C, Feng R, Meng T, Peng W, Song J, et al. CXCR4 regulates macrophage M1 polarization by altering glycolysis to promote prostate fibrosis. Cell Communication and Signaling. 2024 Sep 26;22(1):456.

39. MT2A protein expression summary - The Human Protein Atlas [Internet]. [cited 2025 May 8]. Available from: https://www.proteinatlas.org/ENSG00000125148-MT2A

40. ARG1 protein expression summary - The Human Protein Atlas [Internet]. [cited 2025 May 8]. Available from: https://www.proteinatlas.org/ENSG00000118520-ARG1

41. ERRFI1 protein expression summary - The Human Protein Atlas [Internet]. [cited 2025 May 8]. Available from: https://www.proteinatlas.org/ENSG00000116285-ERRFI1

42. C3 protein expression summary - The Human Protein Atlas [Internet]. [cited 2025 May 8]. Available from: https://www.proteinatlas.org/ENSG00000125730-C3

43. Rautou PE, Tatsumi K, Antoniak S, Owens AP, Sparkenbaugh E, Holle LA, et al. Hepatocyte Tissue Factor Contributes to the Hypercoagulable State in a Mouse Model of Chronic Liver Injury. J Hepatol. 2016 Jan;64(1):53–9.

44. CRP protein expression summary - The Human Protein Atlas [Internet]. [cited 2025 May 8]. Available from: https://www.proteinatlas.org/ENSG00000132693-CRP

45. Wu L, Yan J, Bai Y, Chen F, Zou X, Xu J, et al. An invasive zone in human liver cancer identified by Stereo-seq promotes hepatocyte–tumor cell crosstalk, local immunosuppression and tumor progression. Cell Res. 2023 Aug;33(8):585–603.

46. Birts CN, Barton CH, Wilton DC. Catalytic and non-catalytic functions of human IIA phospholipase A2. Trends in Biochemical Sciences. 2010 Jan 1;35(1):28–35.

Supplementary Figures

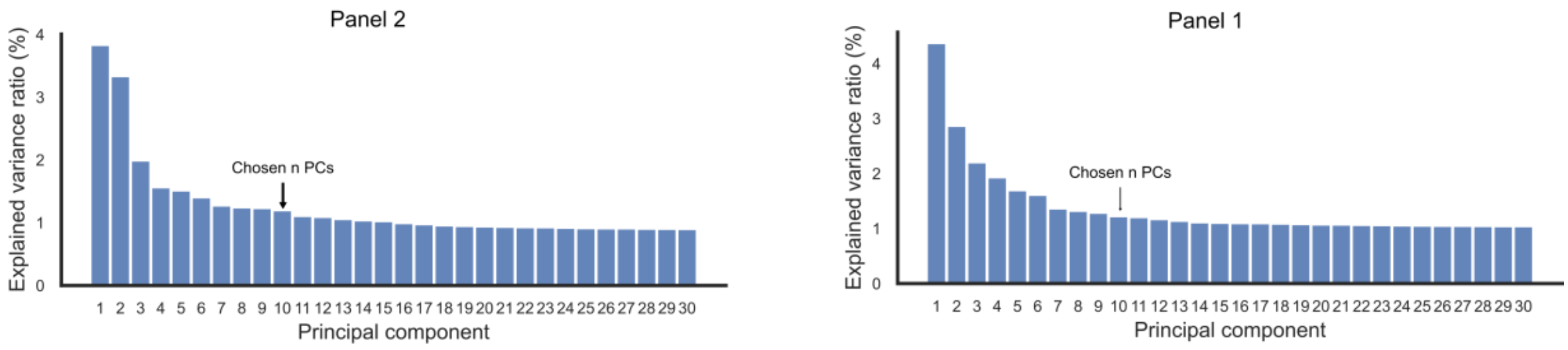

Supplemental Figure 1: PCA selection for Panel 2 and Panel 1. Principal component analysis (PCA) explained variance for Panel 2 and Panel 1. Barplots show the variance explained by the first 30 PCs, with arrows indicating the selected number of PCs used for downstream analysis.

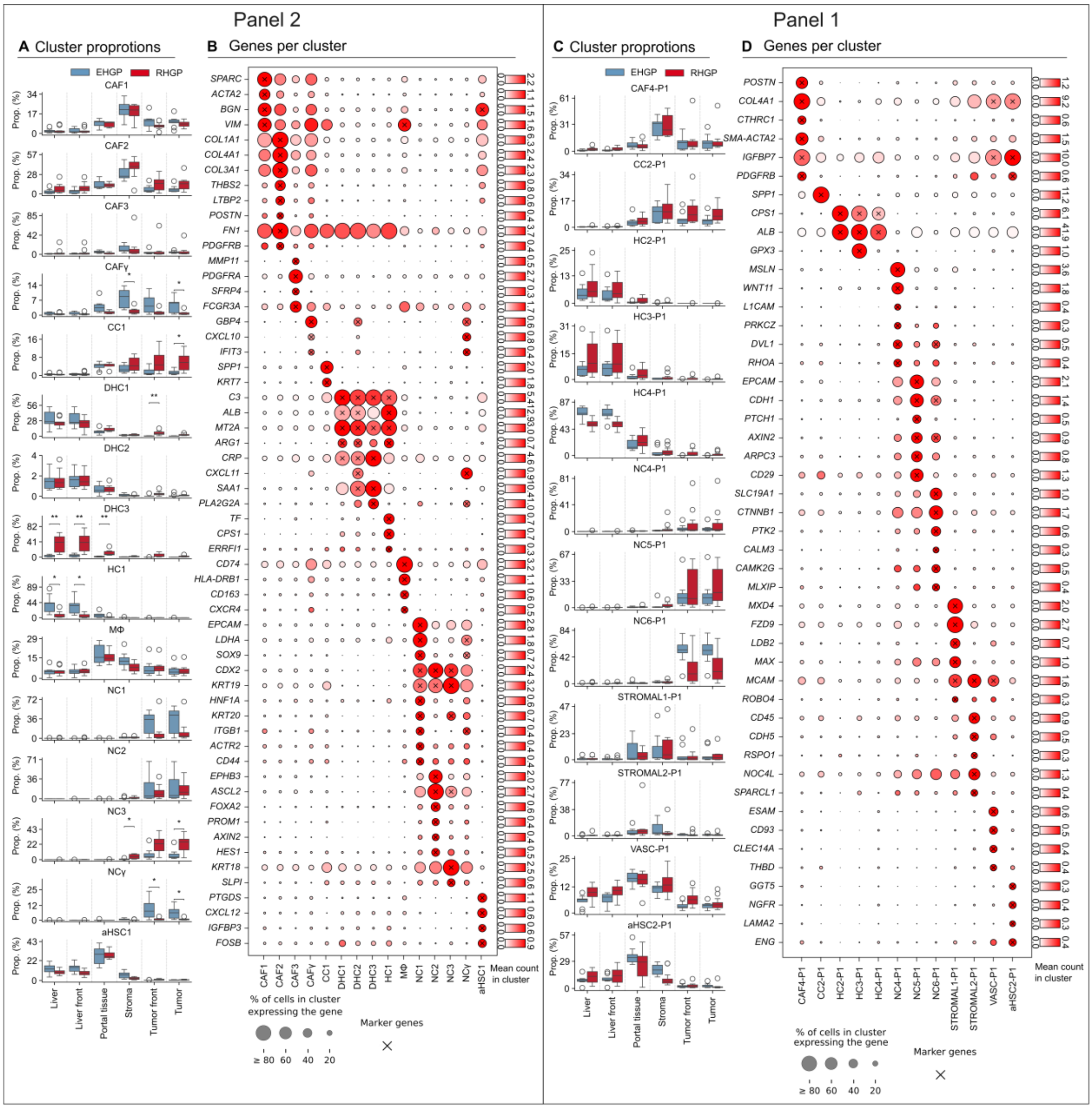

Supplemental Figure 2: Cell cluster proportions by region and gene expression across clusters. The clusters showed gene expression signatures associated with vascular cells (VASC), cancer-associated fibroblasts (CAF), activated hepatic stellate cells (aHSC), cholangiocytes (CC), stromal cells (STROMAL), hepatocytes (HC), Neoplastic cells expressing downstream IFNγ genes (NCγ), macrophages (MΦ), and damaged hepatocytes (DHC). (**A**) The proportion of cell clusters from Panel 2 compared between growth patterns in different regions. (**B**) Dotplot showing marker genes for each cluster; color bars indicate the mean read count for each gene. For example, cells in the DHC3 cluster (**B**) contain, on average, 10.4 reads of SAA1. (**C**–**D**) Equivalent plots for cells and genes from Panel 1. Clusters from Panel 1 are labeled with the suffix "-P1."

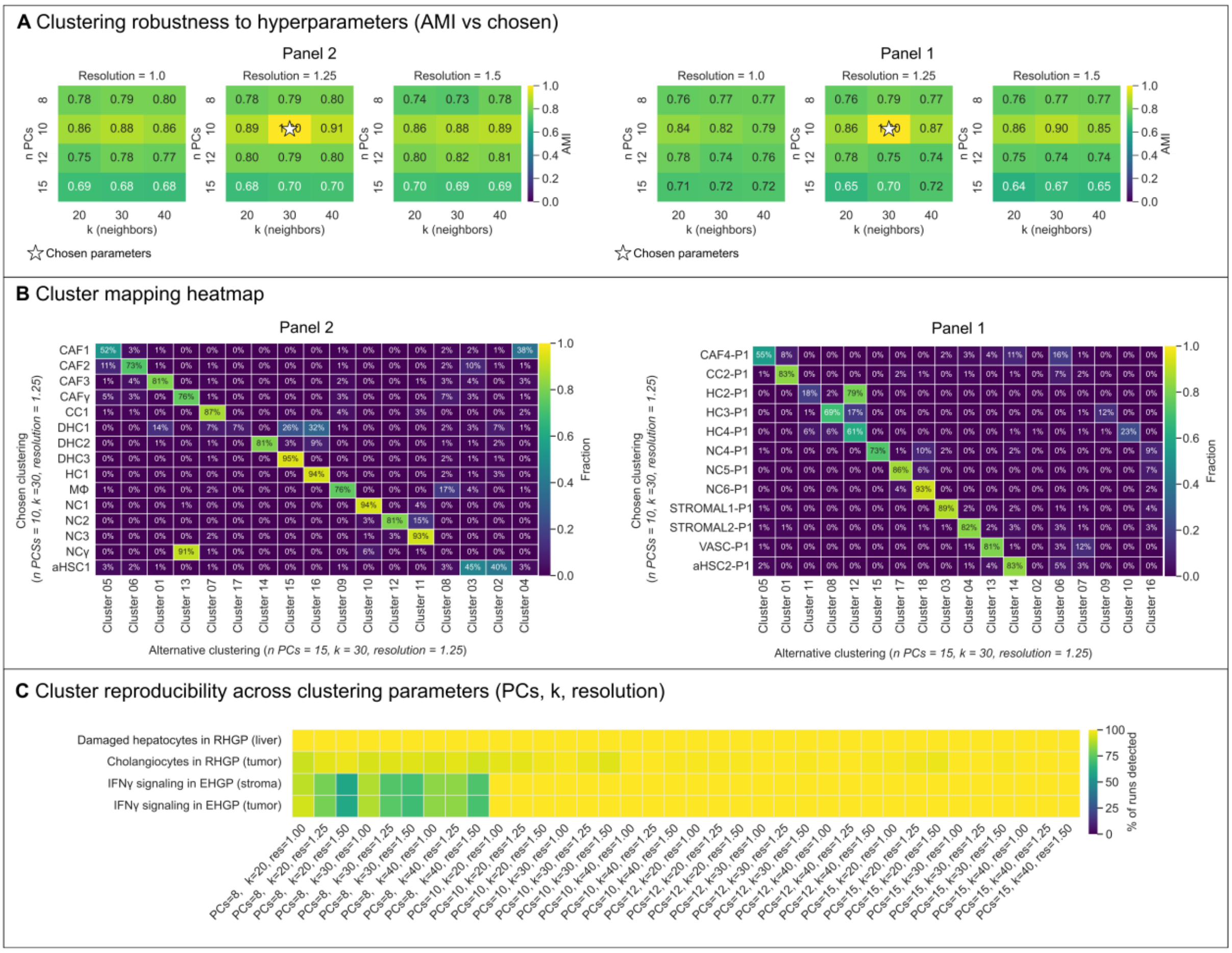

Supplemental Figure 3: Clustering robustness across hyperparameter settings. (**A**) Heatmaps show the Adjusted Mutual Information (AMI) between clusterings obtained under different combinations of number of PCs, neighborhood size (*k*), and resolution parameters, relative to the chosen configuration (indicated by the star). Clustering results were generally stable across *k* and resolution values, whereas AMI decreased modestly when using a larger number of PCs. For each hyperparameter combination, Louvain clustering was run 100 times and the resulting partitions were merged using hierarchical clustering (Methods). (**B**) Cluster-mapping heatmaps for Panel 1 and Panel 2 comparing the selected clustering with an alternative parameter setting (higher number of PCs). The overall cluster structure is maintained, and differences mainly reflect splitting or merging of existing clusters rather than a wholesale reorganization. (**C**) For each clustering configuration (columns), Louvain was run 100 times. Each row shows a cell type (cluster) that met two criteria in a given run: (i) at least two of its marker genes were detected, and (ii) the cluster was enriched in the specified region and growth pattern, based on higher per-patient cluster proportions (one-sided Mann–Whitney U test, $p < 0.05$). Colors indicate the percentage of runs in which the cluster was recovered. Marker genes: damaged hepatocytes (*SAA1*, *CRP*, *PLA2G2A*); cholangiocytes (*KRT7*, *SPP1*); IFNγ-signaling cells (*CXCL10*, *CXCL11*, *GBP4*, *IFIT3*).

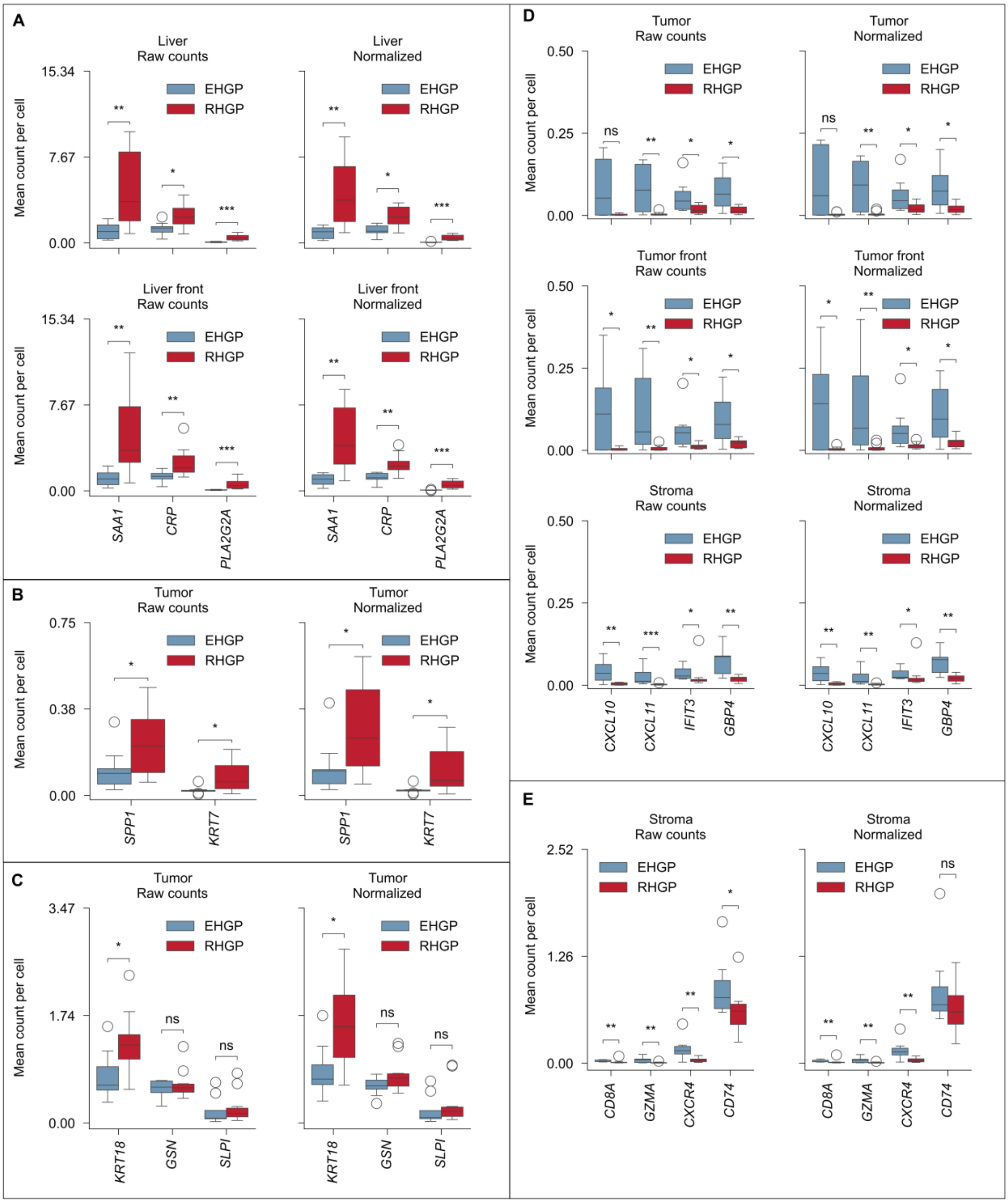

Supplemental Figure 4: Mean read count comparison with sample-wise normalization. To ensure that mean read count comparisons were not biased by sample-specific factors such as tissue quality, each analysis was complemented with a normalized version. Instead of comparing the samples' raw mean count (across cells) of a chosen gene, the mean counts were normalized to the mean total read count. This adjustment was to compensate for differences in overall tissue, preventing samples of lower quality (overall lower counts) from being systematically underrepresented. Panels (**A**–**E**) show comparisons of mean counts using raw counts (left) and normalized counts (right) for genes associated

with: (**A**) damaged hepatocytes, (**B**) cholangiocytes, (**C**) neoplastic cells, (**D**) interferon-γ, and (**E**) cytotoxic T-cells.

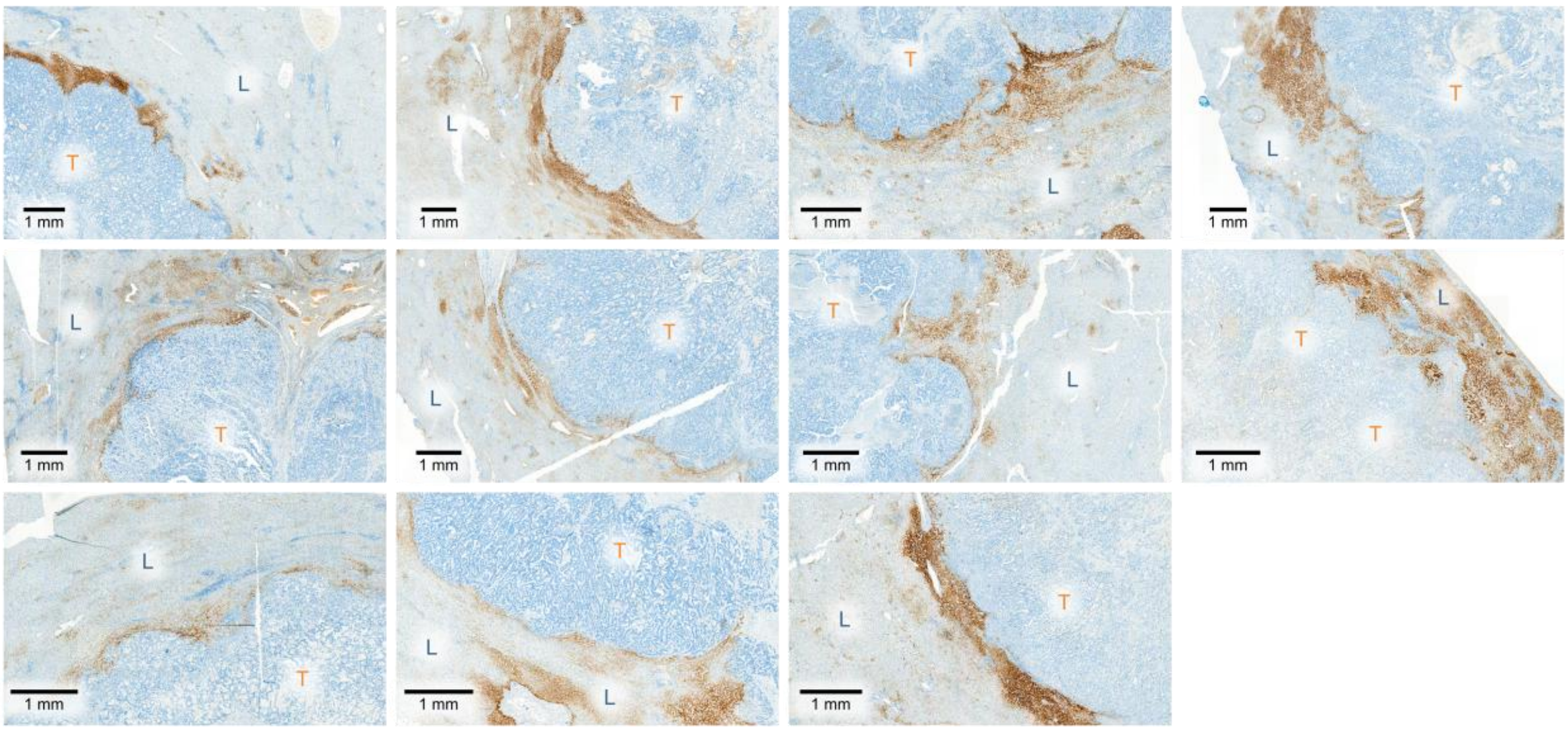

Supplemental Figure 5: *SAA1* Immunohistochemical staining in eleven RHGP colorectal liver metastases. Representative *SAA1* immunohistochemical staining (DAB, hematoxylin counterstain) in eleven RHGP cases, demonstrating expression extending millimeters into adjacent liver tissue beyond the tumor–liver interface. L indicates liver and T indicates tumor.

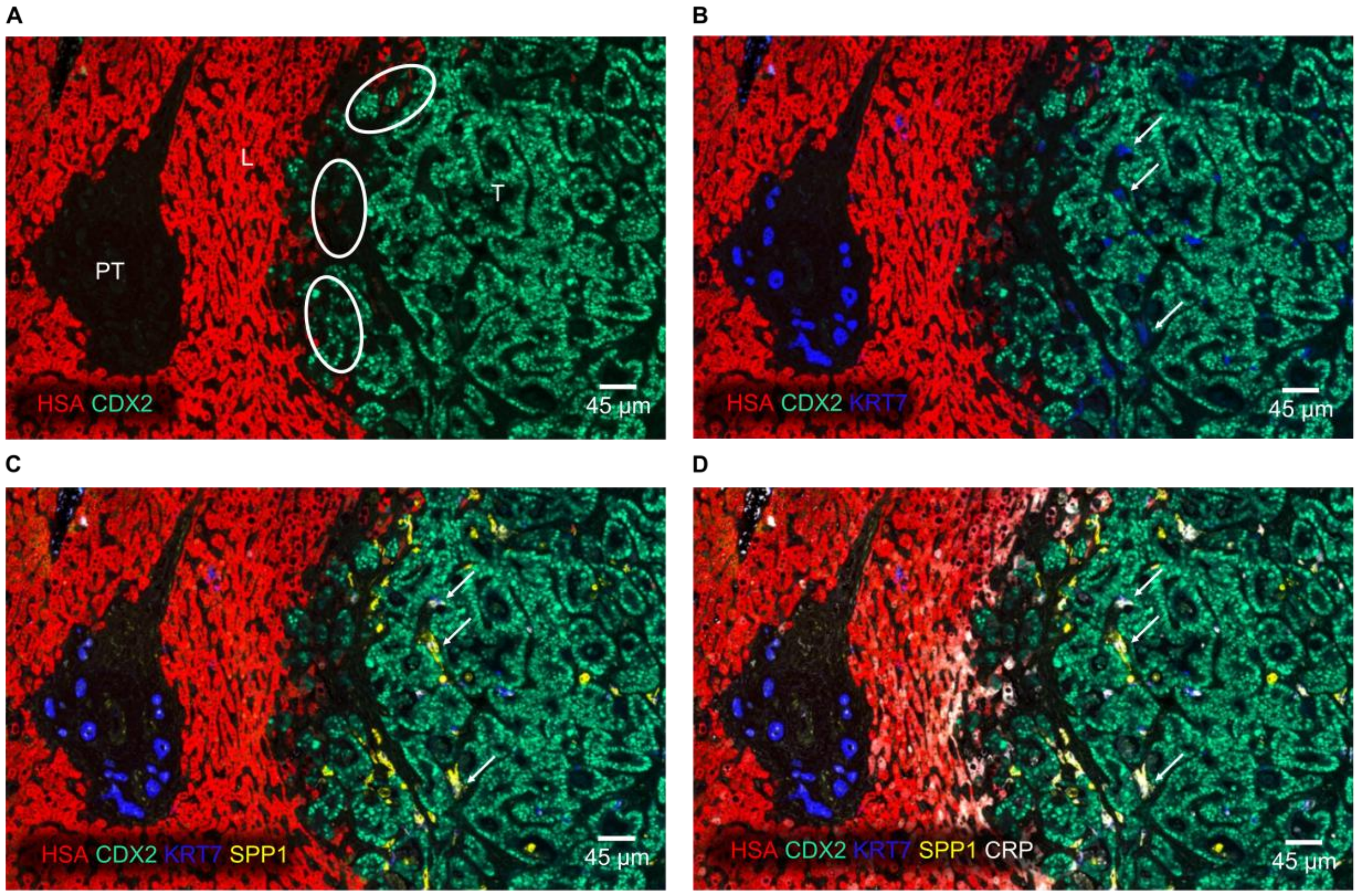

Supplemental Figure 6: Co-opted hepatocyte and cholangiocyte alike phenotypes in an RHGP metastasis revealed by COMET multiplex immunofluorescence. COMET (Lunaphore) multiplex immunofluorescence imaging of the tumor–liver interface showing liver tissue with HSA^{+} hepatocytes (red) and tumor tissue with CDX2 $^{+}$ cancer cells (nuclear green signal). (**A**) Coopted hepatocytes are clearly visible at the interface, forming hybrid solid structures with adjacent cancer cells (examples highlighted with oval annotations). L, T, and PT denote liver, tumor, and portal tract. (**B**) Cytokeratin7–positive (KRT7, blue) and SPP1-positive (SPP1, yellow) cells are observed at a distance from the tumor–liver interface, representing coopted hepatocytes that have lost HSA expression and acquired cholangiocytelike characteristics (**B–C**). Three such cells are indicated by white arrows. These cells also express CRP (white, **D**), an acute phase protein normally derived from hepatocytes.

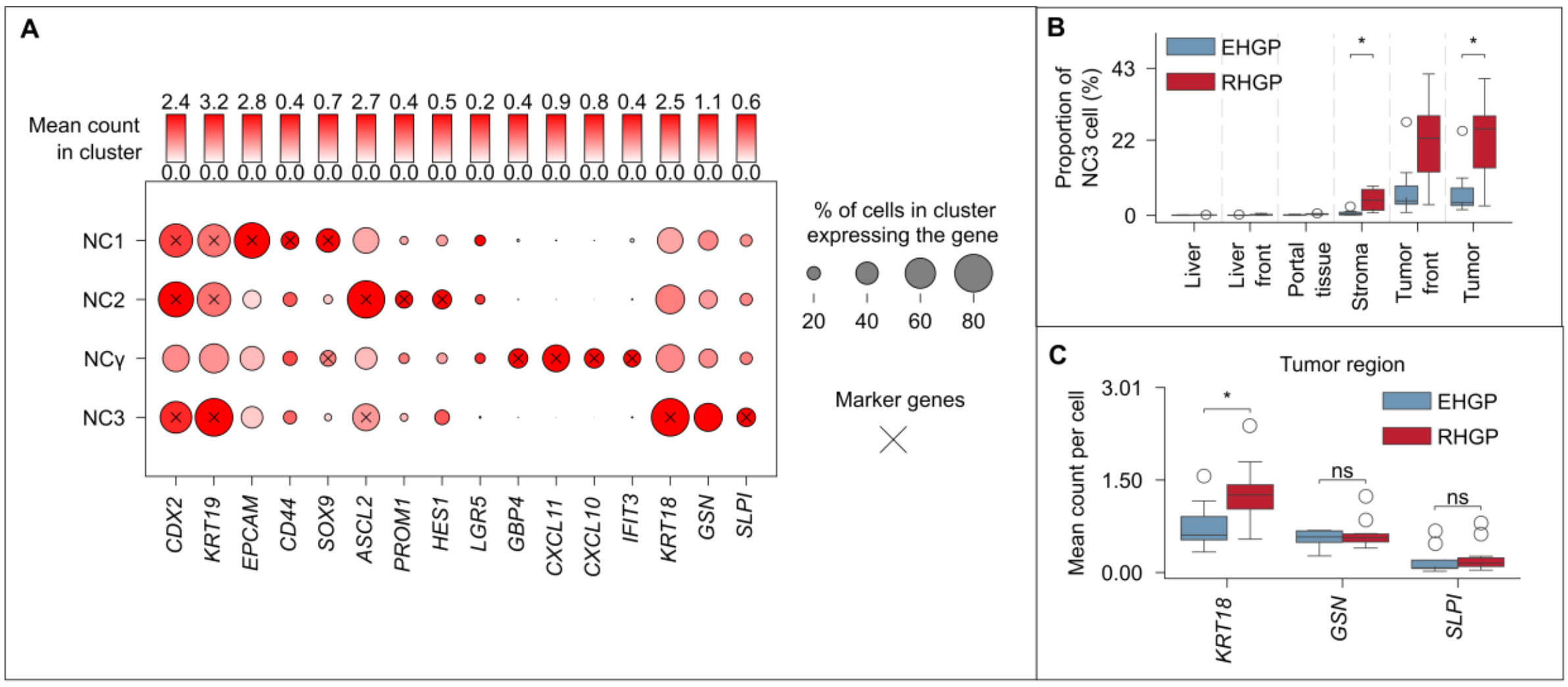

Supplemental Figure 7: A cluster of neoplastic cells was found in a larger proportion in samples with an RHGP. (**A**) Selected marker genes for each of the four neoplastic cell (NC) clusters. (**B**) NC3 cells, with a higher read count of *KRT18, GSN*, and *SLPI*, were found in a larger proportion of samples with an RHGP. (**C**) Mean read count (across cells) of *KRT18*, *GSN*, and *SLPI* in the tumor region compared between RHGP and EHGP samples.

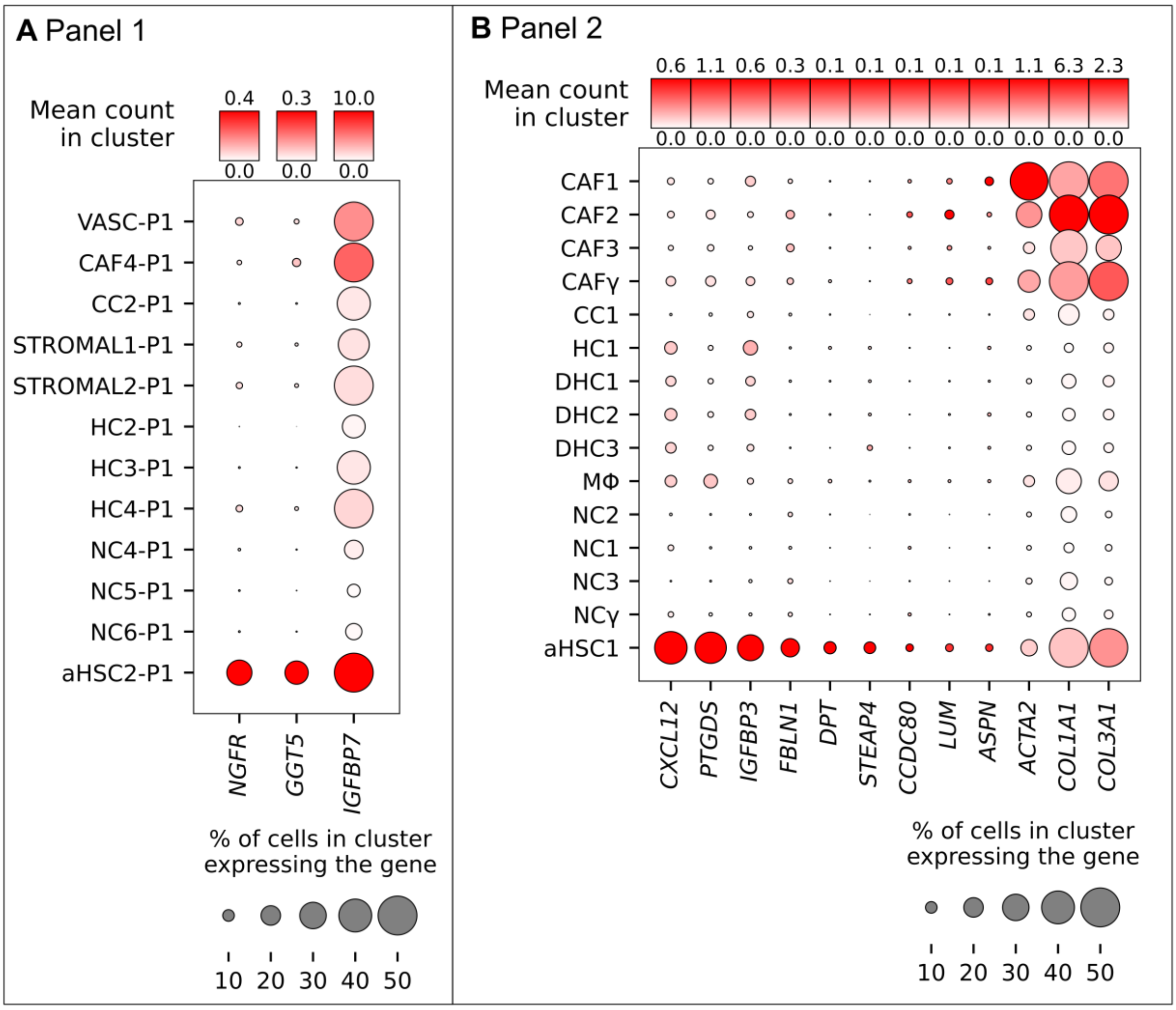

Supplemental Figure 8: Distribution of hepatic stellate cell-related genes across clusters. Genes are extracted from Payen et al. (see reference 40 in main text).

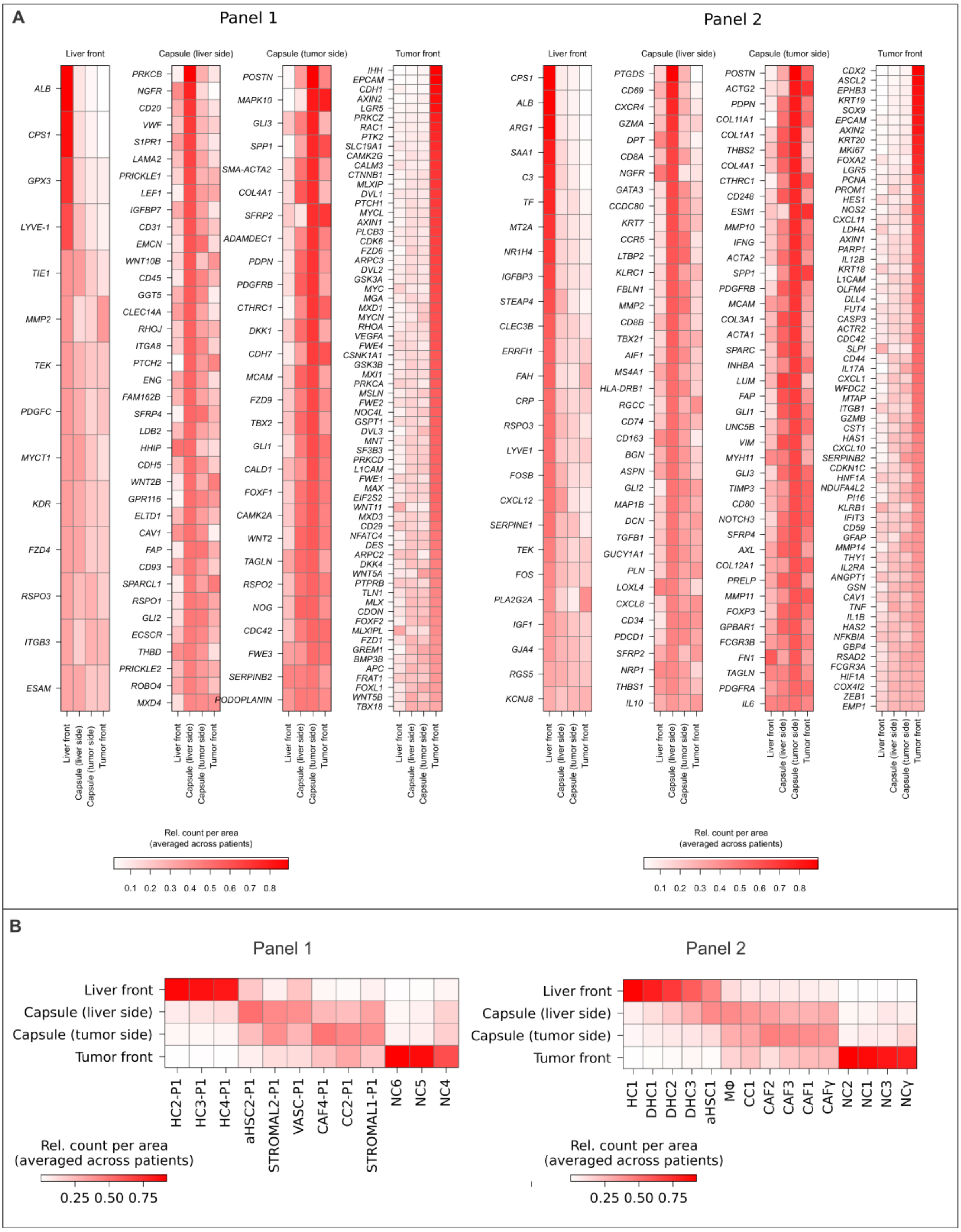

Supplemental Figure 9: Region probability score per gene reveals gene expression zonation of the capsule in EHGP. (**A**) EHGP-labeled lesions were divided into four regions: Liver front, capsule (liver side), capsule (tumor side), and tumor front. The heatmap shows the mean (across samples) relative read count per region area. (**B**) Relative count of clustered cells per region (averaged across EHGP samples).
